# Blimp-1 integrates alarmin signals in ILC2s and drives proinflammatory functions required for type 2 immunity

**DOI:** 10.1084/jem.20250781

**Published:** 2026-03-31

**Authors:** Patrycja M. Forster, Alexandra Preußer, Hiroshi Yano, Divija Deshpande, Wen Zhang, Anita Kowalczyk, Laura Velleman, Magdalena O. Dubik, Jazib Uddin, Elizabeth R. Emanuel, Pierre S. Leclère, Richard Mertens, Xuemei Gao, Michael Kofoed-Branzk, Anja E. Hauser, Hans-Willi Mittrücker, Andreas Diefenbach, David Artis, Christoph S.N. Klose

**Affiliations:** 1 Department of Microbiology, Infectious Diseases and Immunology, Charité – Universitätsmedizin Berlin, Corporate Member of Freie Universität Berlin and Humboldt-Universität zu Berlin, Berlin, Germany; 2 Jill Roberts Institute for Research in Inflammatory Bowel Disease, Weill Cornell Medicine, Cornell University, New York, NY, USA; 3 Friedman Center for Nutrition and Inflammation, Weill Cornell Medicine, Cornell University, New York, NY, USA; 4 Allen Discovery Center for Neuroimmune Interactions, Weill Cornell Medicine, Cornell University, New York, NY, USA; 5 Division of Gastroenterology and Hepatology, Joan and Sanford I. Weill Department of Medicine, Weill Cornell Medicine, Cornell University, New York, NY, USA; 6 Immunology and Microbial Pathogenesis Program, Weill Cornell Medicine, Cornell University, New York, NY, USA; 7 Deutsches Rheuma-Forschungszentrum (DRFZ), an Institute of the Leibniz Association, Berlin, Germany; 8 Department of Rheumatology and Clinical Immunology, Charité – Universitätsmedizin Berlin, Corporate Member of Freie Universität Berlin and Humboldt-Universität zu Berlin, Berlin, Germany; 9 Department of Immunology, University Medical Center Hamburg-Eppendorf, Hamburg, Germany; 10 Hamburg Center for Translational Immunology, University Medical Center Hamburg-Eppendorf, Hamburg, Germany; 11 Cluster of Excellence ImmunoPreCept, Charité - Universitätsmedizin Berlin, Berlin, Germany; 12 Department of Microbiology and Immunology, Weill Cornell Medicine, Cornell University, New York, NY, USA; 13 Parker Institute for Cancer Immunotherapy, Weill Cornell Medicine, Cornell University, New York, NY, USA

## Abstract

Type 2 immunity has evolved to protect against worms but becomes harmful when activated during allergic inflammation. Group 2 innate lymphoid cells (ILC2s) drive type 2 responses by rapidly secreting IL-5 and IL-13. The alarmins, IL-25, IL-33, and TSLP activate ILC2s and are linked to allergic diseases. However, how alarmins connect to the transcriptional networks driving type 2 effector functions remains elusive. Here, we performed RNA sequencing of ILC2s deficient in IL-25, IL-33, or TSLP pathway and identified the transcription factor Blimp-1 as an IL-33–regulated gene in ILC2s. While Blimp-1 was dispensable for ILC2 development, this transcription factor was required for type 2 cytokine production, driving eosinophilia or promoting worm expulsion. Blimp-1 deficiency resulted in reduced IRF4 expression, while *Irf4*-deficient ILC2s showed diminished Blimp-1 and IL-33 receptor expression, revealing a reciprocal Blimp-1–IRF4 circuit downstream of the IL-33 receptor. These findings expose the Blimp-1–IRF4 axis as an alarmin-regulated transcriptional network controlling ILC2 effector functions required for type 2 immunity.

## Teaser

Forster et al. identify the transcriptional network controlling effector functions of ILC2s downstream of the IL-33 receptor. They found that the Blimp-1–IRF4 axis regulates IL-5 and IL-13 production in ILC2s, driving either worm expulsion or eosinophilia during allergic lung inflammation.

## Graphical Abstract

**Figure:**
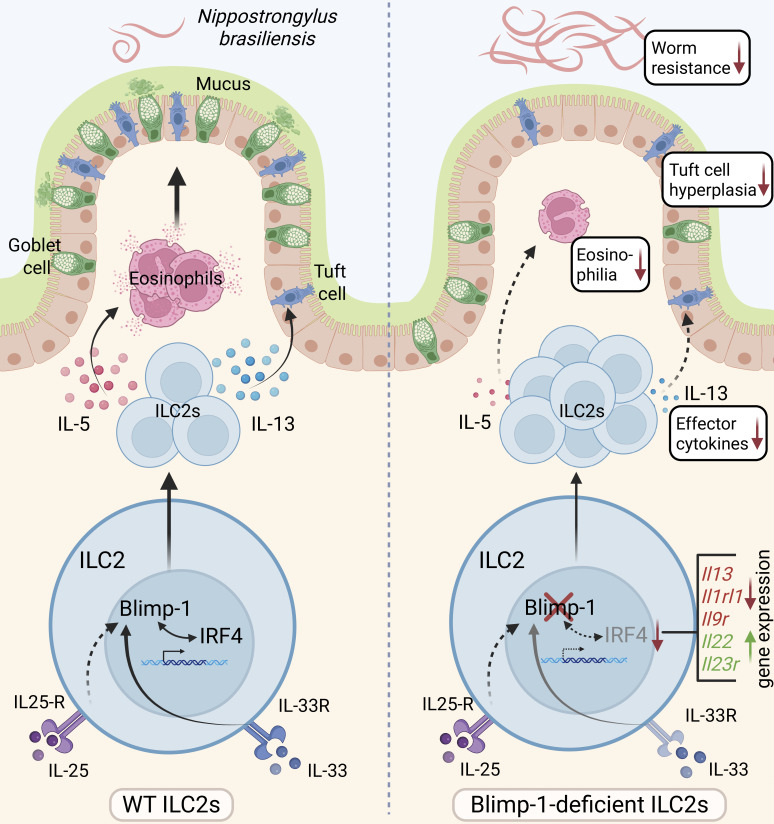

## Introduction

To mount an efficient immune response at barrier surfaces upon pathogenic encounters, the immune system is often triggered by danger signals, such as alarmins, resulting in the activation of tissue-resident immune cells. The alarmins IL-25, IL-33, and thymic stromal lymphopoietin (TSLP) are danger signals mainly secreted by epithelial and stromal cells in response to infection and tissue damage (Stanbery et al., 2022). After release, the alarmins activate group 2 innate lymphoid cells (ILC2s), resulting in rapid secretion of type 2 cytokines, such as IL-5 and IL-13, which promote eosinophil recruitment, mucus production, and tissue repair, triggering worm expulsion or allergic inflammation (Kabata et al., 2020; Neill et al., 2010; Stanbery et al., 2022; Vivier et al., 2018; Zaiss et al., 2024). IL-33 has been associated with allergic diseases in both experimental mouse models and human genome-wide association studies, particularly in the context of allergic asthma, making it a promising molecular target for therapeutic intervention (Li et al., 2015; Moffatt et al., 2010; Torgerson et al., 2011). Furthermore, IL-33 and TSLP levels were elevated in asthmatic patients, and these two alarmins synergize to drive type 2 inflammation (Li et al., 2018; Toki et al., 2020). Finally, the TSLP-blocking antibody tezepelumab was approved for treatment of severe asthma in humans (Menzies-Gow et al., 2021). In parallel, IL-25 produced primarily by tuft cells upon sensing succinate from helminths or protozoans (Gerbe et al., 2016; Howitt et al., 2016; Schneider et al., 2018; von Moltke et al., 2016) promotes the differentiation of a subset of ILC2s with migratory capacity, termed inflammatory ILC2s (iILC2s) (Burrows et al., 2025; Huang et al., 2015). While IL-25–driven responses are critical for worm expulsion, it has also been shown that IL-33 signaling is required for worm resistance and can promote iILC2 differentiation as well, albeit to a lesser extent (Flamar et al., 2020; Hung et al., 2013). Although the transcription factor BATF is required for iILC2 differentiation (Miller et al., 2020), the transcriptional networks regulating ILC2 function and their link to alarmin signals are poorly understood.

The development and functional programming of ILC2s are governed by a network of transcription factors, including GATA-3, ID2, ROR-α, BCL11B, and GFI-1 (Furusawa et al., 2013; Halim et al., 2012; Hoyler et al., 2012; Klein Wolterink et al., 2013; Moro et al., 2010; Wong et al., 2012; Yu et al., 2015). These transcription factors are often expressed in several ILC subsets and uncommitted ILC progenitors, and their depletion typically results in an almost complete absence of ILC2s (Harly et al., 2019; Klose et al., 2014). While this reflects the importance of these transcription factors for ILC2 development, the molecular targets and signaling pathways regulated by these transcription factors are incompletely understood. In contrast, alarmin signals are dispensable for ILC2 development but required for their activation, suggesting other transcription factors could be involved in this process (Ricardo-Gonzalez et al., 2018; Topczewska et al., 2023). Identifying these transcriptional regulators of ILC2 effector functions and defining how they interface with alarmin signaling is therefore critical for understanding the molecular basis of ILC2 activation.

B lymphocyte–induced maturation protein 1 (Blimp-1), encoded by the gene *Prdm1*, is a broadly expressed transcription factor with essential roles in several cell types, including immune cell populations. It is required for the terminal differentiation of B cells into antibody-secreting plasma cells (Shaffer et al., 2002), the maturation of natural killer (NK) cells (Kallies et al., 2011), and effector T cell maturation (Cretney et al., 2011). For T cell skewing from undifferentiated Th0 cells, Blimp-1 is required for T helper type 2 (Th2) cell differentiation and type 2 cytokine production, in part by regulating the lineage-specifying transcription factor GATA-3 (He et al., 2020). In ILC2s, Blimp-1 has been linked to IL-10 production, and morpholino-mediated Blimp-1 KO reduced IL-10 expression in these cells (Howard et al., 2021).

In this study, we identify Blimp-1 as a transcription factor regulated by IL-33 signaling. While Blimp-1 was dispensable for ILC2 development, it was necessary for type 2 effector cytokine production in steady state and under inflammatory conditions. Conditional deletion of *Prdm1* in ILC2s did not prevent proliferation or differentiation into iILC2 during worm infection; however, these cells failed to terminally differentiate into cytokine-producing effector cells. As a consequence, ILC2-restricted Blimp-1–deficient mice exhibited impaired eosinophilia and epithelial activation, leading to delayed worm expulsion and diminished allergic lung inflammation. Transcriptomic profiling of *Prdm1*-deficient ILC2s revealed that Blimp-1 regulates *Irf4* expression, a transcription factor critical for ILC2 activation and anti-helminth immunity (Honma et al., 2008; Mohapatra et al., 2016). Vice versa, *Irf4*-deficient ILC2s exhibited reduced *Prdm1* and IL-33 receptor chain (*Il1rl1*) expression, exposing the IL-33–Blimp-1–IFN regulatory factor 4 (IRF4) axis as a regulatory network controlling ILC2 activation and effector function. Collectively, our study identifies Blimp-1 as a key transcription factor downstream of the IL-33 receptor that, together with IRF4, enables ILC2 responses to combat worm infections and orchestrates effective type 2 immunity.

## Results

### Alarmin signals regulate distinct pathways in ILC2s

**Figure S1.**
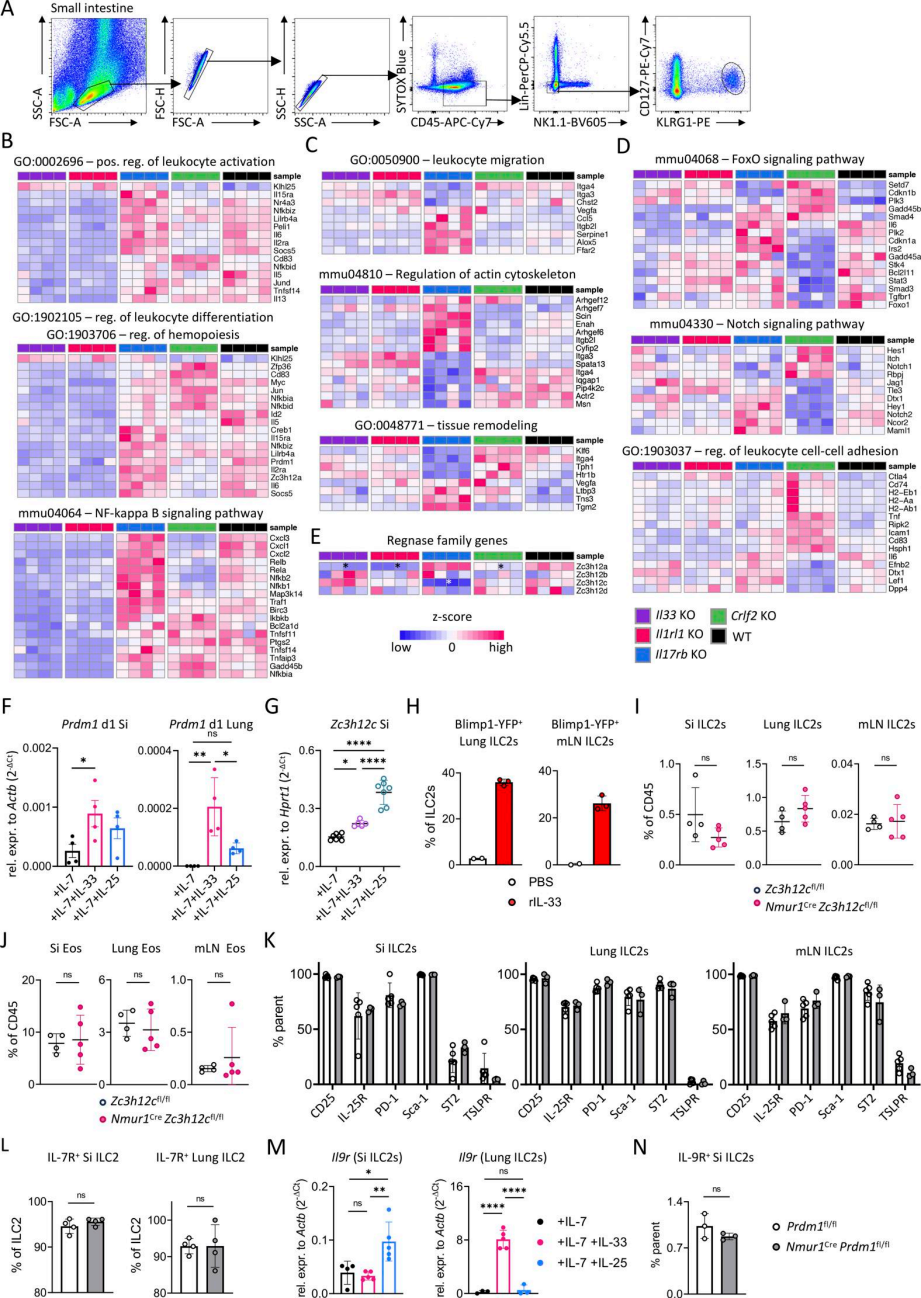
**Alarmin-regulated pathways and characterization of** ***Prdm1*** **- and** ***Zc3h12c*** **-deficient mice** **.** Related to Figs. 1 and 2. **(A)** Gating strategy for sort purification of small intestinal ILC2 prior to bulk RNA-seq. **(B–D)** Heatmaps of genes for the indicated pathways. **(E)** Heatmap of the different Regnase genes; * indicates significant regulation (P < 0.05). **(F)** Relative expression of *Prdm1* in stimulated small intestinal and lung ILC2s. Cells were stimulated for 1 day with IL-7, IL-7 + IL-33, or IL-7 + IL-25. Data are representative of two independent experiments (*n* = 4 mice per group). **(G)** Relative expression of *Zc3h12c* in stimulated small intestinal ILC2s. Cells were stimulated for three days with IL-7, IL-7 + IL-33, or IL-7 + IL-25. Data are representative of two independent experiments (*n* = 5–7 mice per group). **(H)** Quantification of Blimp-1–YFP–expressing ILC2s from the lungs and mLN after stimulation with PBS (Ctrl) or rIL-33; *n* = 2–3 mice per group. **(I and J)** Quantification of flow cytometric analysis of ILC2s (I) and eosinophils (Eos) (J) from *Zc3h12c*^fl/fl^ and *Nmur1*^Cre^*Zc3h12c*^fl/fl^ mice from the indicated organs. **(K)** Quantification of flow cytometric analysis showing different ILC2 markers in ILC2s from *Prdm1*^fl/fl^ and *Nmur1*^Cre^*Prdm1*^fl/fl^ mice from the indicated organs. Data are representative of two independent experiments (*n* = 4–5 mice per group). **(L)** Quantification of flow cytometric analysis showing IL-7R expression in ILC2s from *Prdm1*^fl/fl^ and *Nmur1*^Cre^*Prdm1*^fl/fl^ mice from the indicated organs. **(M)** Relative expression of *Il9r* in stimulated small intestinal (Si) and lung ILC2s. Cells were stimulated for 1 day with IL-7, IL-7 + IL-33, or IL-7 + IL-25; *n* = 4–5 mice per group. **(N)** Quantification of flow cytometric analysis of IL-9R–expressing lung ILC2s from *Prdm1*^fl/fl^ and *Nmur1*^Cre^*Prdm1*^fl/fl^ mice; *n* = 3 mice per group. Mean ± SD; Student’s *t* test (H–J, L, and N); or one-way ANOVA (F, G, K, and M); ns, nonsignificant; *P < 0.05; **P < 0.01; ****P < 0.0001.

Alarmins are pivotal cytokines stimulating ILC2 activation at barrier surfaces. To dissect the specific roles of individual alarmins in regulating ILC2 functions, we performed bulk RNA-sequencing (RNA-seq) on sort-purified intestinal ILC2s from mice deficient in the IL-25 (*Il17rb*^−/−^ mice), the IL-33 (*Il33*^−/−^ and *Il1rl1*^−/−^ [gene for the IL-33 receptor chain ST2] mice) or the TSLP (*Crlf2*^−/−^ mice) signaling pathway, alongside C57BL/6 WT controls (Fig. S1 A).

**Figure 1.**
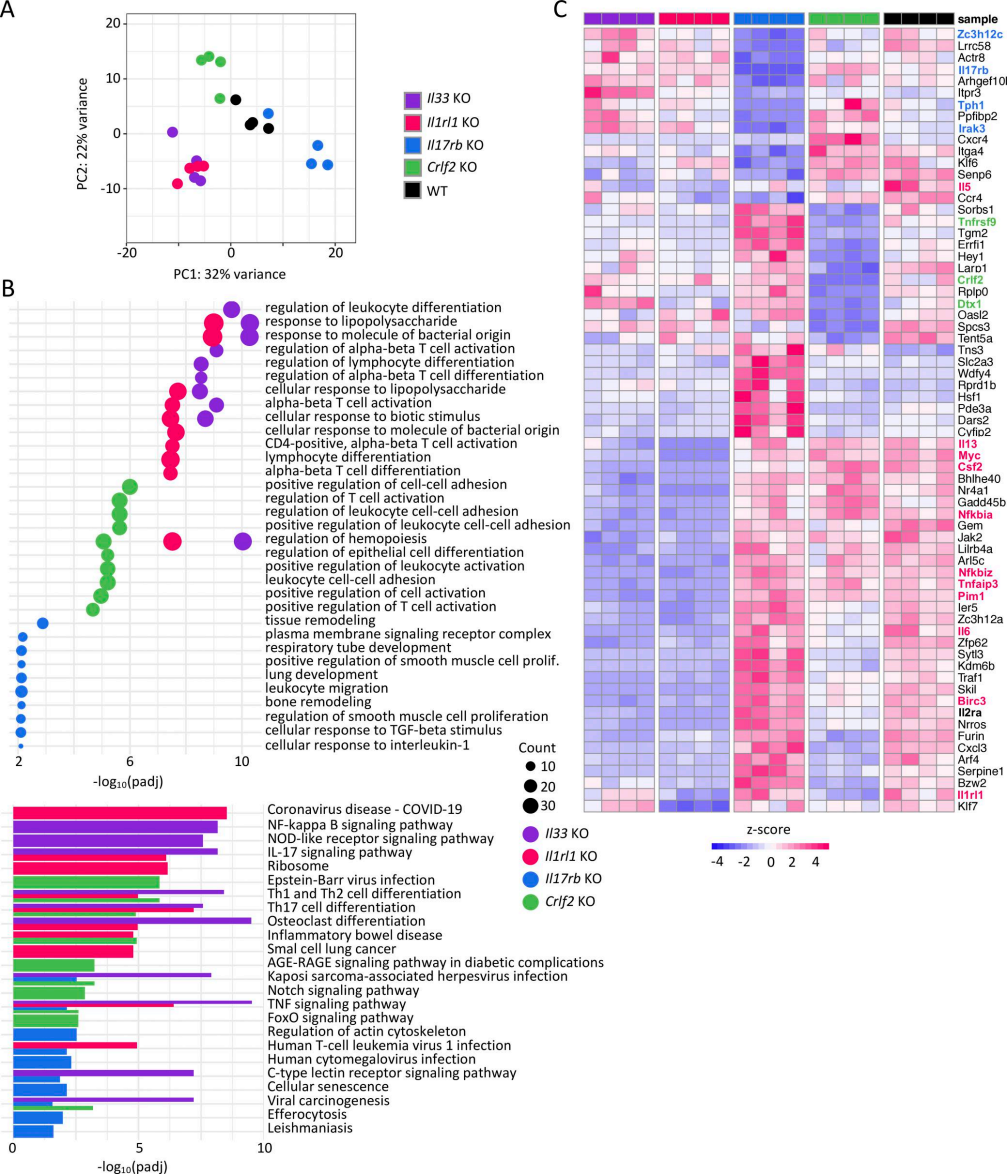
**Transcriptional profiling of ILC2s from various strains of alarmin-deficient mice. (A)** PCA of bulk RNA-seq of small intestinal ILC2s isolated from *Il33* KO, *Il1rl1* KO, *Il17rb* KO, *Crlf2* KO, and WT mice. **(B)** GO and KEGG pathway analysis of differentially regulated genes in the KOs from B. **(C)** Heatmap of the top differentially regulated genes in the KOs. Genes specific to each KO are indicated with different colors. Genes of interest are marked in red. **(A–C)***n* = 4 mice per group.

The principal component analysis (PCA) revealed distinct clustering of WT and alarmin signaling-deficient ILC2s, suggesting that different alarmins elicit unique gene regulatory programs in ILC2s (Fig. 1 A). As expected, *Il33*^−/−^ and *Il1rl1*^−/−^ ILC2s clustered closely together, consistent with the shared defects in the IL-33 signaling pathway (Fig. 1 A). Gene Ontology (GO) and Kyoto Encyclopedia of Genes and Genomes (KEGG) pathway analysis identified distinct sets of differentially alarmin-regulated genes. *Il33*^−/−^ and *Il1rl1*^−/−^ ILC2s showed enrichment of pathways related to “positive regulation of leukocyte activation,” “regulation of leukocyte differentiation,” and “NF-κB signaling,” while “leukocyte migration,” “regulation of actin cytoskeleton,” and “tissue remodeling” were identified in *Il17rb*^−/−^ ILC2s. In *Crlf2*^−/−^ ILC2s, we detected altered “Foxo signaling,” “Notch signaling,” and “regulation of leukocyte cell–cell adhesion” pathways (Fig. 1 B and Fig. S1, B–D).

Heatmap analysis of differentially regulated genes highlighted genotype-specific clustering (Fig. 1 C). Strikingly, under steady state conditions, the effector cytokines *Il5*, *Il13*, and *Csf2* were consistently downregulated in *Il33*^−/−^ and *Il1rl1*^−/−^ ILC2s but not in *Il17rb*^−/−^ or *Crlf2*^−/−^ mice. This finding extends previous studies assigning an important role for the IL-33 signaling pathways in ILC2 for B1 cells, eosinophils, and type 2 immunity (Jarick et al., 2022; Troch et al., 2024). IL-33 signaling also controlled the cell cycle genes *Myc*, *Birc3*, and *Pim1* and NF-κB pathway genes *Nfkbia* and *Nfkbiz* and *Tnfaip3* (Schneider et al., 2018). Interestingly, *Irak3*, a downstream modulator of the Myd88 signaling pathway, was downregulated in *Il17rb*^−/−^ but upregulated in *Il33*^−/−^, *Il1rl1*^−/−^, and *Crlf2*^−/−^ ILC2s, suggesting a potential link between alarmin signaling pathways and *Irak3*. Loss of IL-25R in ILC2s led to the downregulation of genes involved in migration (*Ccr4*, *Itga4*, and *Itga3*), serotonin production (*Tph1*), and tissue remodeling (*Vegfa*), highlighting the function of IL-25 in regulation of ILC2 homing, migration, and resolution of inflammation (Fig. 1 C and Fig. S1, B–D). Additionally, the gene *Zc3h12c*, which encodes an RNase involved in inflammatory cytokine mRNA degradation (Regnase-3), was among the most downregulated genes in *Il17rb*^−/−^ ILC2s, suggesting a potential role of IL-25 signaling in posttranscriptional control of ILC2 effector functions via *Zc3h12c* expression (von Gamm et al., 2019). In contrast, Regnase-1, encoded by the *Zc3h12a* gene, was dependent on the other alarmin signaling pathways IL-33 and TSLP (Fig. S1 E). *Crlf2*^−/−^ ILC2s exhibited altered expression of the Notch target genes (*Notch1*, *Rbpj*, and *Dtx1)*, the activation-induced receptor *Tnfrsf9*, and the *Il2ra* chain (Fig. 1 C and Fig. S1 D) (Flamar et al., 2020; Huang et al., 2015). Taken together, our transcriptional analysis identifies both, shared and distinct gene programs regulated by IL-25, IL-33, and TSLP in ILC2s. These findings provide insights into how alarmin signals shape ILC2 heterogeneity and fate decisions, revealing potential mechanisms underlying the functional plasticity of ILC2s in type 2 immunity.

### Differentially expressed genes (DEGs) include *Prdm1 and Zc3h12c,* which are dispensable for ILC2 development

**Figure 2.**
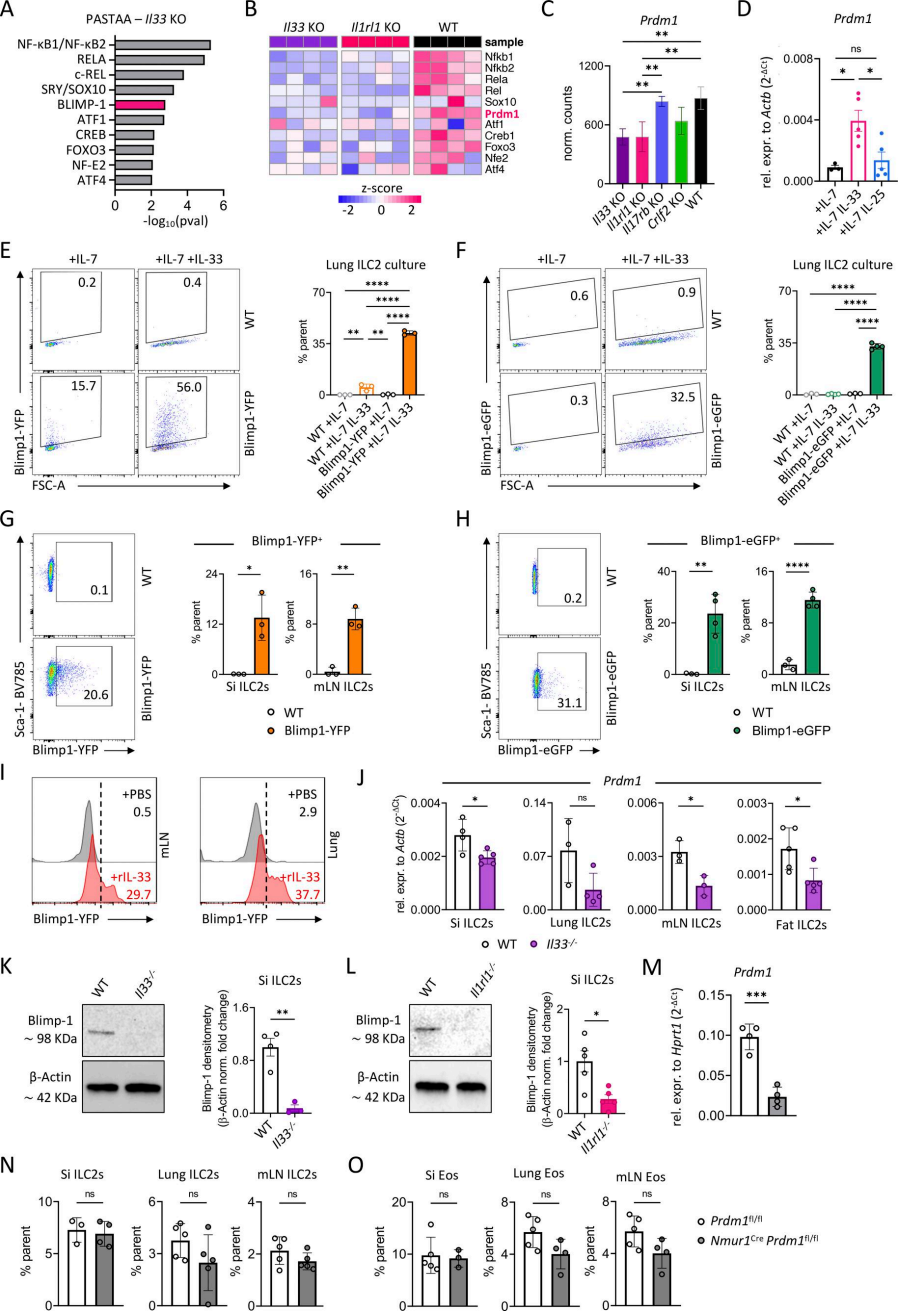
**IL-33 regulates Blimp-1 in ILC2s. (A)** Predicting associated transcription factors from annotated affinities (PASTAA) of differentially regulated genes from small intestinal ILC2s of *Il33* KO vs. WT mice, showing potential transcription factor regulation. **(B)** Heatmap of transcription factors differentially regulated in small intestinal ILC2s of *Il33* KO and *Il1rl1* KO vs. WT mice. **(C)** Normalized counts of the gene *Prdm1* across all KOs. **(A–C)***n* = 4 mice per group. **(D)** Relative expression of *Prdm1* in stimulated small intestinal ILC2s. Cells were stimulated for 3 days with IL-7, IL-7 + IL-33, or IL-7 + IL-25. Data are representative of two independent experiments; *n* = 3–5 mice per group. **(E and F)** Flow cytometric plots and quantification of Blimp-1–YFP (E) and Blimp-1–eGFP (F) expression in lung ILC2s following *in vitro* IL-7 and IL-33 or IL-7 only stimulation, assessed in WT and Blimp-1–YFP or Blimp-1–eGFP mice. **(G and H)** Flow cytometric plots and quantification of Blimp-1 expression in ILC2s from small intestine (Si) and lungs of Blimp-1–YFP (G) or Blimp-1–eGFP (H) reporter mice in steady state. **(E–H)***n* = 3–4 mice per group. **(I)** Histograms of Blimp-1–YFP–expressing ILC2s from the lungs and mLN after stimulation with PBS (Ctrl) or rIL-33, *n* = 2–3 mice per group. **(J)** Relative expression of the gene *Prdm1* in ILC2s from indicated tissues. Data are representative of two independent experiments; *n* = 3–4 mice per group. **(K and L)** Representative western blot from *Il33*^−/−^ and WT mice (K) and from *Il1rl1*^−/−^ and WT mice (L) and quantification of K and L. Data are representative of two independent experiments; *n* = 3–5 mice per group. **(M)** Relative expression of *Prdm1* in small intestinal ILC2s from *Prdm1*^fl/fl^ and *Nmur1*^Cre^*Prdm1*^fl/fl^ mice; *n* = 4 mice per group. **(N and O)** Quantification of flow cytometric analysis of ILC2s (N) and eosinophils (Eos) (O) from *Nmur1*^Cre^*Prdm1*^fl/fl^ and littermate control (*Prdm1*^fl/fl^) mice from the indicated organs. Data are representative of two independent experiments; *n* = 3–5 mice per group. Mean ± SD; Student’s *t* test (G, H, J, K, L, M, N, and O) or one-way ANOVA (C–F); ns, nonsignificant; *P < 0.05; **P < 0.01; ***P < 0.001; ****P < 0.0001. Source data are available for this figure: SourceData F2.

Given the distinct transcriptional profiles observed across the different alarmin KO, we next sought to dissect the downstream signaling cascades driving these differences (Fig. 1, A–C). Since IL-33 signaling controlled effector cytokines and downstream transcriptional programs, we performed predicting associated transcription factors from annotated affinities (PASTAA) analysis to identify candidate transcription factors regulating these gene sets (Roider et al., 2009). Consistent with IL-33 signaling via the Myd88 adapter mediating NF-κB translocation, several downstream mediators of this pathway, including NF-κB1/NF-κB2, RELA, and c-REL, were identified. In addition, our analysis identified the transcription factor Blimp-1 as a predicted regulator of gene sets differentially expressed in ILC2s (Fig. 2 A). Expression analysis confirmed that *Prdm1* mRNA (encoding for Blimp-1) was consistently downregulated in *Il33*^−/−^ and *Il1rl1*^−/−^ ILC2s compared with *Il17rb*^−/−^ and *Crlf2*^−/−^ ILC2s, suggesting that ILC2s need constant IL-33 signals to maintain Blimp-1 expression (Fig. 2, B and C). To directly test whether alarmin stimulation induces *Prdm1* expression, sort-purified ILC2s were cultured *in vitro* and stimulated with IL-7 and IL-33 or IL-25. IL-33 stimulation resulted in robust upregulation of *Prdm1*, which was significantly higher compared with IL-25–stimulated ILC2s (Fig. 2 D). Increased *Prdm1* expression was already detectable on day one after stimulation and also detectable in lung ILC2s following IL-33 stimulation (Fig. S1 F). Conversely, *Zc3h12c* (encoding Regnase-3) expression was induced after IL-25 treatment (Fig. S1 G).

To further corroborate these transcriptional findings, we used two different Blimp-1 reporter lines, Blimp-1–YFP and Blimp-1–eGFP. We performed flow cytometry analysis on ILC2s from different organs at steady state and, additionally, following an *in vitro* stimulation. In line with our mRNA data, stimulation of sort-purified lung ILC2s with IL-7 and IL-33 induced strong Blimp-1 expression (Fig. 2, E and F). We detected Blimp-1 expression in ILC2s in all organs examined, although small intestinal ILC2s, known as potent type 2 cytokine producers, had the highest expression, (Hoyler et al., 2012) (Fig. 2, E–H). Moreover, administration of recombinant IL-33 *in vivo* resulted in pronounced upregulation of Blimp-1 in both lung and mesenteric lymph nodes (mLNs) ILC2s (Fig. 2 I and Fig. S1 H), further supporting the role of IL-33 in inducing Blimp-1 expression.

We confirmed and extended these findings by measuring *Prdm1* mRNA by qPCR in ILC2s from various tissues. The consistent reduction of *Prdm1* in *Il33*^−/−^ ILC2s argues that the Blimp-1 regulation by IL-33 signaling is broadly happening in ILC2s and is not restricted to ILC2s from specific organs (Fig. 2 J). On the protein level, western blot analysis demonstrated a strong reduction of Blimp-1 in sort-purified *Il33*^−/−^ and *Il1rl1*^−/−^ ILC2s from the small intestine compared with WT controls (Fig. 2, K and L).

**Figure S2.**
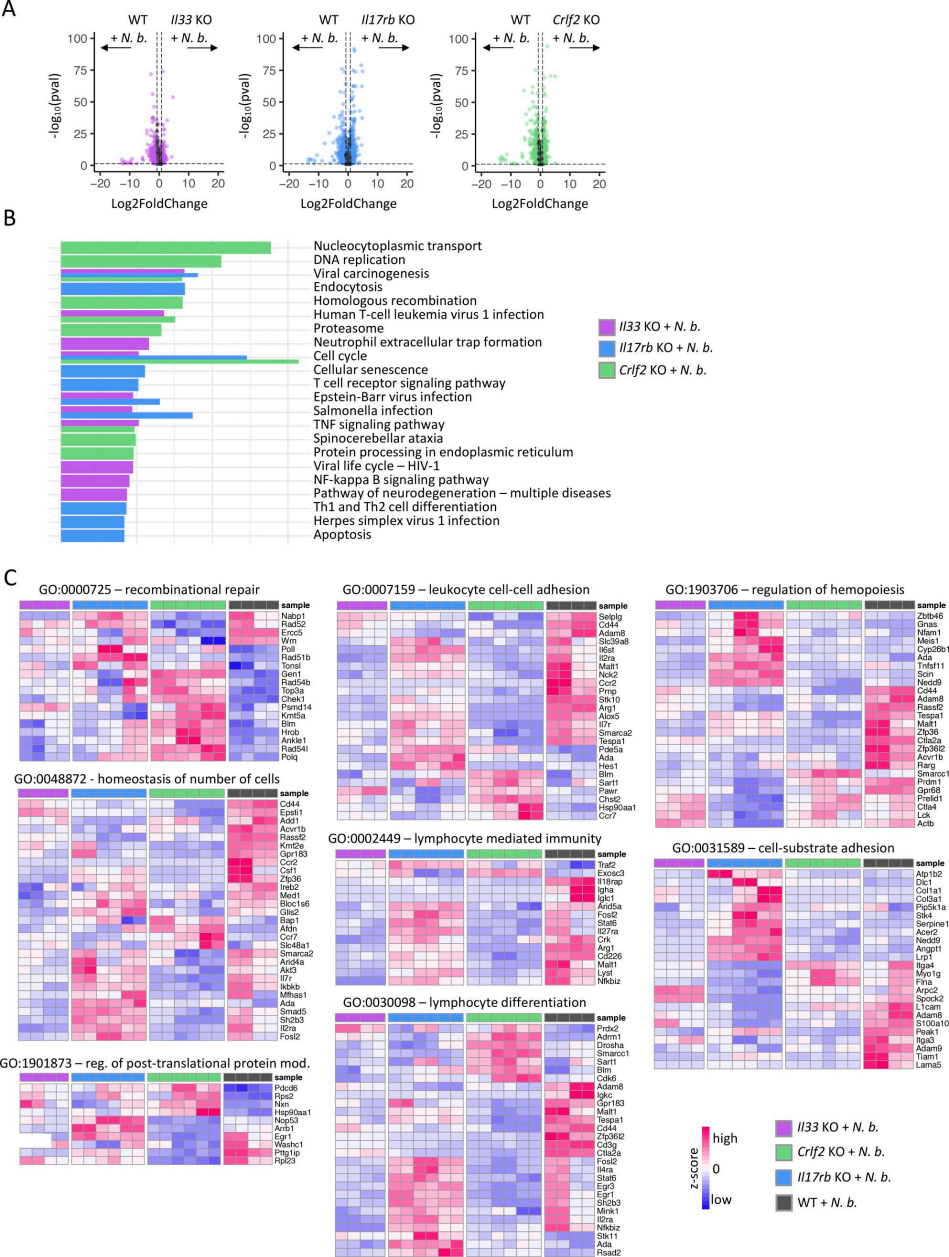
**Transcriptomic analysis of alarmin-deficient ILC2s in** ***N. brasiliensis*** **infection** **.** Related to Fig. 3. **(A)** Volcano plots of DEGs from the indicated infected KOs compared with infected WT mice. Mice were infected with *N. brasiliensis* for 7 days. **(B)** KEGG pathway analysis of infected KOs compared with infected WT mice. **(C)** Heatmaps of genes for pathways shown in Fig. 3 A. **(A–C)***n* = 2–3 mice per group showing technical replicates.

Together, our analyses identified *Zc3h12c* (Regnase-3) and *Prdm1* (Blimp-1) as potential downstream modulators of IL-25 and IL-33 signaling, respectively. To explore the function of Regnase-3 and Blimp-1 in ILC2s, we genetically ablated these genes by crossing *Nmur1*^Cre^ to *Zc3h12c*^fl/fl^ and *Prdm1*^fl/fl^ mice. Flow cytometry analysis did not reveal significant differences between ILC2s in *Nmur1*^Cre^*Zc3h12c*^fl/fl^ mice and *Zc3h12c*^fl/fl^ littermate controls. Similarly, eosinophils as target cells of ILC2-derived IL-5 and, therefore, surrogate marker of ILC2 function, were unaffected by the *Zc3h12c* deletion (Fig. S1, I and J). Therefore, we concluded that Regnase-3 is not required for ILC2 homeostasis. Following a similar strategy, we next examined *Nmur1*^Cre^*Prdm1*^fl/fl^ mice. After confirming the successful deletion of *Prdm1* transcripts (Fig. 2 M), we analyzed ILC2s at steady state across several organs, including the small intestine, lung, and mLN. ILC2s developed normally in *Nmur1*^Cre^*Prdm1*^fl/fl^ mice, with no notable difference in the expression of phenotypic ILC2 markers, such as the ST2, IL25R, or TSLP receptor (TSLPR), or in eosinophil proportions compared with *Prdm1*^fl/fl^ littermate control mice (Fig. 2, N and O; and Fig. S1 K). Similarly, *Prdm1*-deficient ILC2s from both the small intestine and the lung showed no differences in IL-7R levels (Fig. S1 L). As ILC2s upregulate the IL-9 receptor (IL-9R) upon activation, enabling the IL-9–dependent autocrine circuit that reinforces ILC2 proliferation and cytokine production (Fig. S2 M), we hypothesized that loss of Blimp-1 might alter *Il9r* expression. However, *Il9r* surface expression was comparable between controls and *Prdm1*-deficient mice, indicating that Blimp-1 is not required for IL-9R regulation in lung ILC2s during steady state (Fig. S2 N). Thus, these conditional KO (cKO) data show that Blimp-1 is dispensable for the development of ILC2s.

Together, our data show that Regnase-3 and Blimp-1 are regulated by IL-25 and IL-33 signaling, respectively, in a context-dependent manner.

### IL-33- and IL-25-signaling-deficient ILC2s fail to induce *Prdm1* expression during worm infection

**Figure 3.**
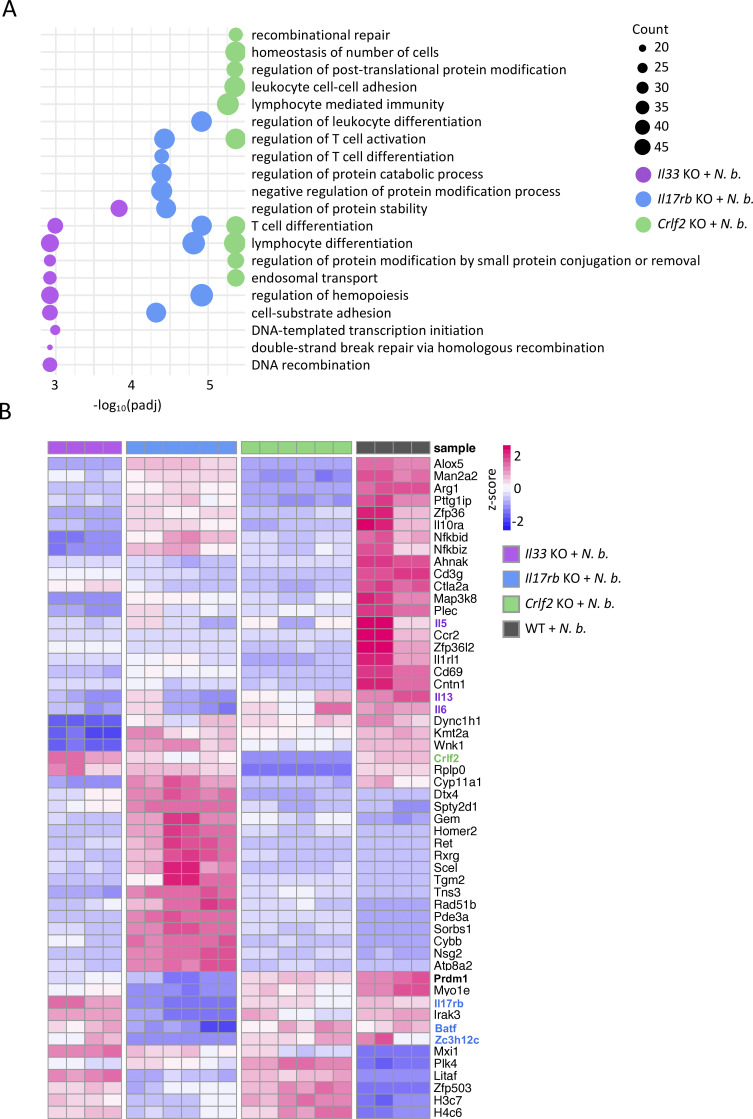
**Blimp-1 fails to be upregulated under inflammatory conditions in alarmin-deficient ILC2s. (A)** GO pathway analysis of differentially regulated genes from bulk RNA-seq of small intestinal ILC2s from *Il33* KO, *Il17rb* KO, and *Crlf2* KO compared with WT mice after *N. brasiliensis* infection. **(B)** Heatmap of the top differentially regulated genes from the different KOs 7 days after *N. brasiliensis* infection. *N*. *b*., *N*. *brasiliensis*. **(A and B)***n* = 2–3 mice per group, showing technical replicates.

Building on our *in vitro* stimulation data showing the induction of *Prdm1* upon IL-33 exposure, we aimed to explore alarmin-mediated gene regulation in the context of type 2 inflammation during worm infection. To this end, we performed bulk RNA-seq on sort-purified intestinal ILC2s from *Il33*^−/−^, *Il17rb*^−/−^, and *Crlf2*^−/−^ mice and C57BL/6 WT controls on day 7 after *Nippostrongylus brasiliensis* (*N. brasiliensis*) infection, a migratory gut-dwelling nematode eliciting a strong ILC2 response. We identified a substantial number of differentially regulated genes and pathways, mirroring the data observed under steady state conditions, further highlighting the importance of the alarmin signaling in ILC2s even in the absence of inflammation (Fig. 3 A and Fig. S2, A–C). However, overall, the overlapping pathways between the different alarmin KOs seem to increase, suggesting a convergence of affected pathways upon infection, as indicated in Fig. 1 B versus Fig. 3 A.

At the gene level, expression of the alarmin receptors was altered under inflammatory conditions (Fig. 3 B). Similarly, genes involved in NF-κB signaling were expressed at lower levels in alarmin-deficient ILC2s, consistent with the triggering of this pathway during inflammation. Extending on our steady state findings, the type 2 effector cytokines were downregulated in all alarmin KO conditions following worm infection, underscoring the dynamic nature of type 2 response. Notably, *Prdm1* was diminished in ILC2s from *Il33*^−/−^ and *Il17rb*^−/−^ mice during worm infection (Fig. 3 B). Moreover, also *Zc3h12c* and the transcription factor *Batf* were selectively downregulated in the *Il17rb*^−/−^ mice, with the reduction in *Batf* expression aligning with published data on its requirement for iILC2s (Miller et al., 2020).

Thus, our findings argue that the expression of Blimp-1 and Regnase-3 strictly require alarmin signals at steady state and during type 2 inflammation. The essential role of IL-25 and IL-33 for ILC2 activation and worm expulsion prompted us to investigate whether ILC2s require Blimp-1 and Regnase-3 for effective worm control.

### Blimp-1–deficient ILC2s fail to promote worm expulsion despite exhibiting a pronounced inflammatory phenotype

**Figure S3.**
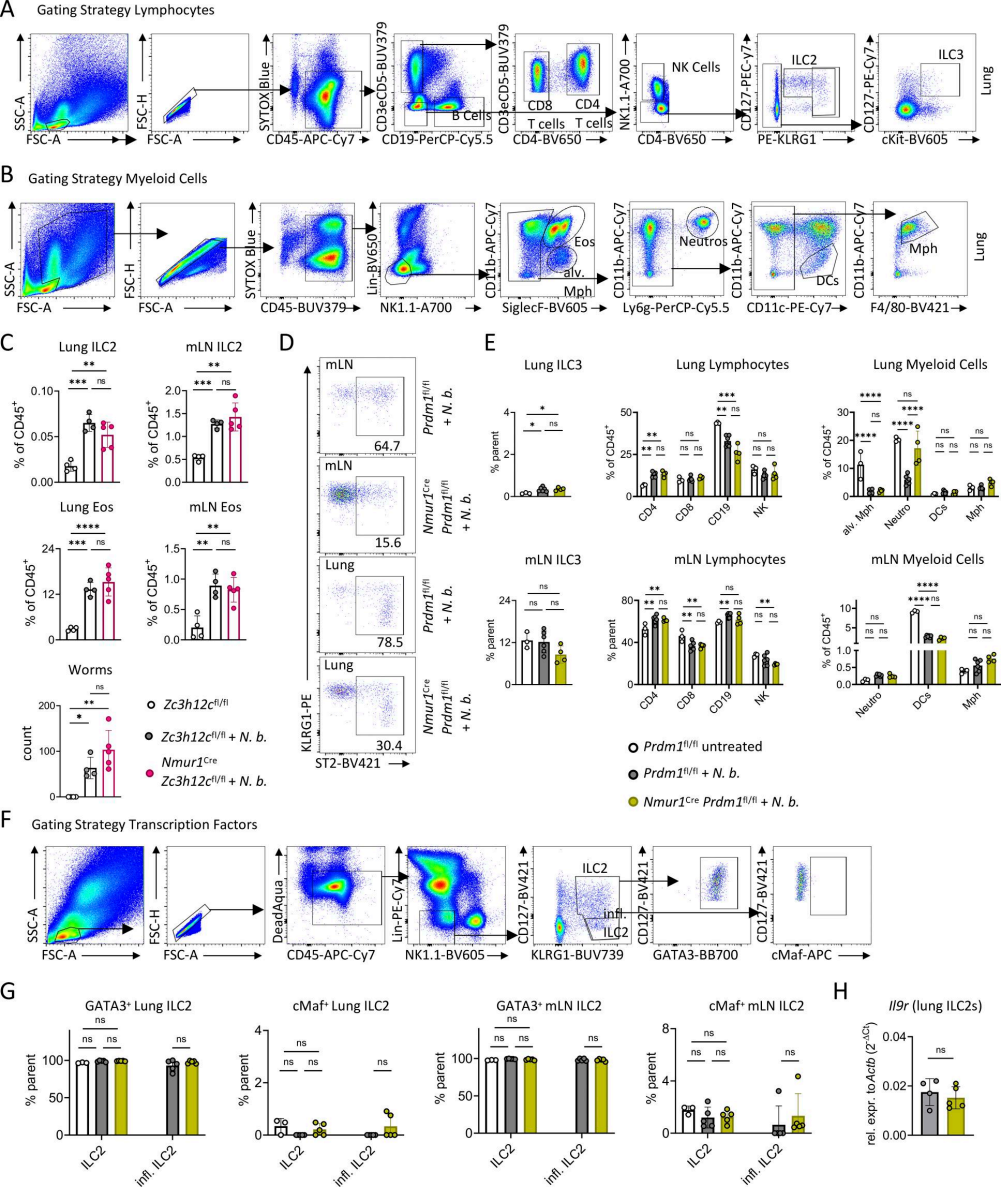
**Analysis of** ***Prdm1*** **- and** ***Zc3h12c*** **-deficient mice during *N. brasiliensis* infection** **.** Related to Fig. 4. **(A)** Gating strategy for lymphocytes shown in Fig. 4 A and Fig. S3, C and E. **(B)** Gating strategy of myeloid cells shown in Fig. 4 C and in Fig. S3, C and E. **(C)** Quantification of flow cytometric analysis of lung and mLN ILC2s and eosinophils (Eos) from untreated and infected *Zc3h12c*^fl/fl^ and infected *Nmur1*^Cre^*Zc3h12c*^fl/fl^ mice. Worm burden of untreated and infected mice. Mice were infected with *N. brasiliensis* for 7 days. Data are representative of two independent experiments; *n* = 4–5 mice per group. **(D)** Flow cytometric plots of ST2 expression (related to Fig. 4 B) in mLN and lung ILC2s from infected *Prdm1*^fl/fl^ and infected *Nmur1*^Cre^*Prdm1*^fl/fl^ mice; *n* = 4–6 mice per group. **(E)** Quantification of flow cytometric analysis of lymphocytes and myeloid cells from naive and infected *Prdm1*^fl/fl^ and infected *Nmur1*^Cre^*Prdm1*^fl/fl^ mice. Data are representative of two independent experiments; *n* = 3–6 mice per group. **(F)** Gating strategy for G. **(G)** Flow cytometric analysis of the expression of transcription factors GATA3 and cMaf in ILC2s from untreated and infected *Prdm1*^fl/fl^ and infected *Nmur1*^Cre^*Prdm1*^fl/fl^ mice (*n* = 3–5 mice per group). **(H)** Relative expression of *Il9r* in lung ILC2s from untreated and infected *Prdm1*^fl/fl^ and infected *Nmur1*^Cre^*Prdm1*^fl/fl^ mice. Mice were infected with *N. brasiliensis* for 7 days (*n* = 4–5 mice per group). *N*. *b*., *N*. *brasiliensis*. Mean ± SD; Student’s *t* test (H) or one-way ANOVA (C, E, and G); ns, nonsignificant; *P < 0.05; **P < 0.01; ***P < 0.001; ****P < 0.0001.

To investigate the function of Blimp-1 and Regnase-3 in ILC2s during a dynamic type 2 response characterized by IL-25 and IL-33 secretion, we infected *Nmur1*^Cre^*Prdm1*^fl/fl^ and *Nmur1*^Cre^*Zc3h12c*^fl/fl^ mice, along with littermate controls, with *N. brasiliensis*. We did not detect significant changes in *Nmur1*^Cre^*Zc3h12c*^fl/fl^ mice under inflammatory conditions, including in ILC2s, eosinophils, or worm burden (Fig. S3, A–C), arguing for a redundant role of Regnase-3 in ILC2s.

**Figure 4.**
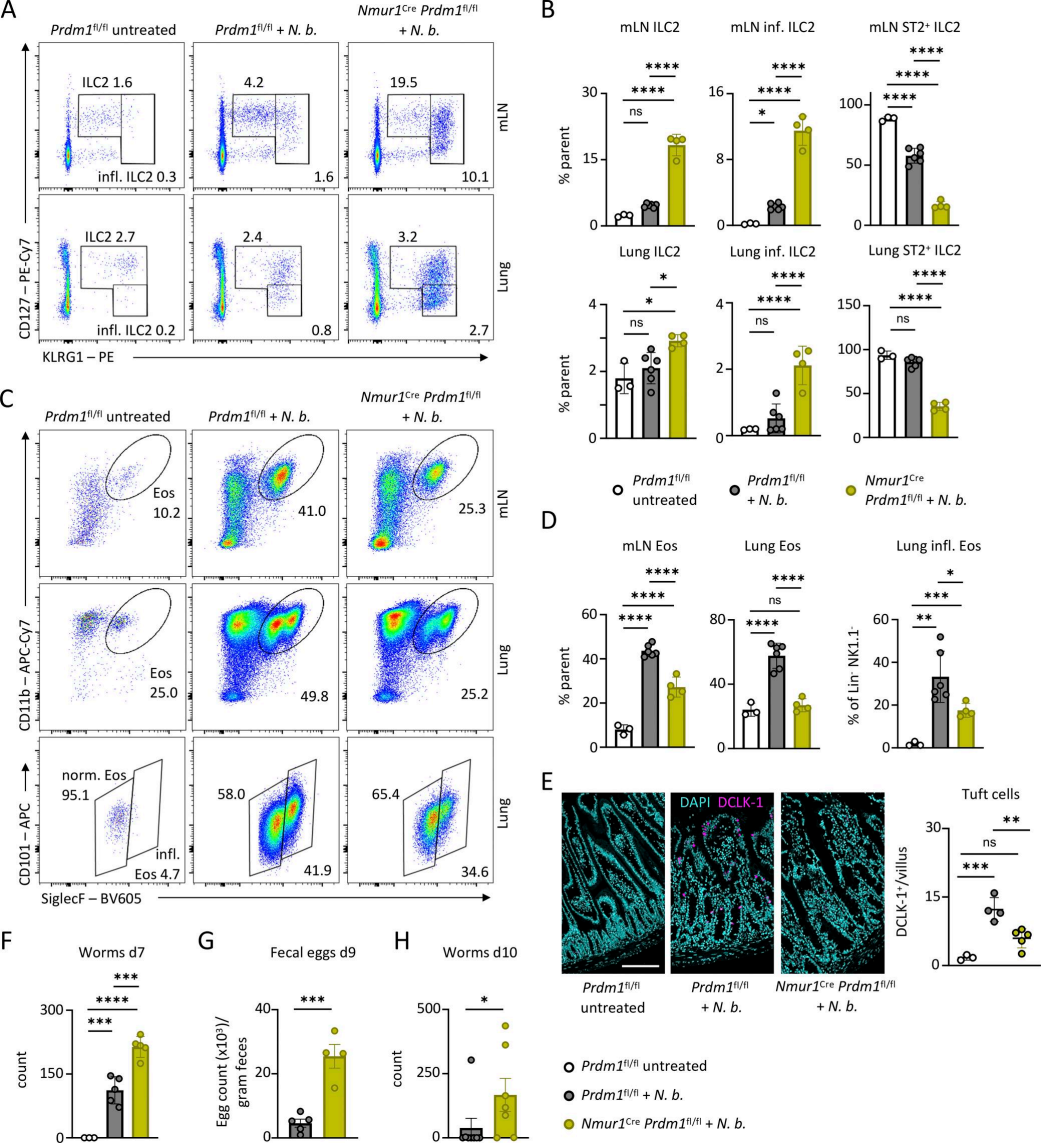
**Blimp-1–deficient ILC2s fail to protect from *N. brasiliensis* infection. (A)** Flow cytometric plots of ILC2s and iILC2s in mLN and lungs from untreated and infected Ctrl (*Prdm1*^fl/fl^) and cKO (*Nmur1*^Cre^*Prdm1*^fl/fl^) mice. Mice were infected with *N. brasiliensis* for 7 days. **(B)** Quantification of A and quantification of ST2 (*Il1rl1*) expression in ILC2s from untreated, infected Ctrl, and infected cKO mice. **(C)** Flow cytometric plots of eosinophils and inflammatory eosinophils in mLN and lungs from untreated, infected Ctrl, and cKO mice. Mice were analyzed 7 days after infection with *N. brasiliensis*. **(D)** Quantification of C in untreated, infected Ctrl, and infected cKO mice. **(E)** Immunofluorescence staining of murine small intestinal sections showing DCLK1^+^ tuft cells (magenta) and DAPI (cyan). Scale bar = 200 µm. Quantification was performed by counting tuft cells per villus. **(F)** Worm burden in the intestine of untreated, infected Ctrl, and infected cKO mice. **(A–F)** Data are representative of two independent experiments (*n* = 3–6 mice per group). **(G)** Number of fecal eggs at day 9 after infection in cKO and littermate Ctrl mice. **(H)** Quantification of *N. brasiliensis* worm burden day 10 after infection. *N*. *b*., *N*. *brasiliensis*. **(G and H)** Data are representative of two independent experiments (*n* = 3–5 mice per group). Mean ± SD; Student’s *t* test (G) or Mann–Whitney U test (Wilcoxon rank-sum test) (H) or one-way ANOVA (B, D, E, and F); ns, nonsignificant; *P < 0.05; **P < 0.01; ***P < 0.001; ****P < 0.0001.

In contrast, Blimp-1–deficient ILC2s exhibited significant alterations in several key parameters (Fig. 4, A and B; and Fig. S3, A, B, D, and E). Following *N. brasiliensis* infection, iILC2s, characterized by CD127 and ST2 downregulation, emerged in control mice. However, the percentage of ILC2 and iILC2 was even higher in *Nmur1*^Cre^*Prdm1*^fl/fl^ mice (Fig. 4, A and B; and Fig. S3 D). Thus, the differentiation and expansion of iILC2s were not impaired by the loss of *Prdm1*. Despite the increased expansion and inflammatory phenotype, ILC2s in these mice were ineffective in mounting a type 2 immune response for worm expulsion. This was reflected by decreased eosinophilia and diminished epithelial response, including tuft cell hyperplasia in *Nmur1*^Cre^*Prdm1*^fl/fl^ mice, which are downstream of IL-5 and IL-13, respectively (Fig. 4, C–E) (Kopf et al., 1996; McKenzie et al., 1999; Nussbaum et al., 2013). The curtailed type 2 response resulted in increased worm burden in the intestine (Fig. 4 F), which was persistent even until day 10 after infection, as indicated by high abundance of fecal eggs on day 9 and the failure to expel the worms until day 10 (Fig. 4, G and H). Although ILC2 function was altered in cKO mice, we did not observe any changes in the transcription factor GATA-3, which is known to be important for ILC2 development and function. Furthermore, Blimp-1 has also been linked to cMaf in regulating IL-10 expression by ILC2s (Howard et al., 2021); however, we did not detect any changes of cMaf under inflammatory conditions (Fig. S3, F and G). Finally, because IL-9R contributes to an autocrine circuit that amplifies ILC2 activation, we assessed *Il9r* expression in *Prdm1*-deficient ILC2s (Fig. S3 H). Consistent with our steady state observations, *Il9r* expression remained unchanged in cKO mice and was not altered upon infection-induced activation.

In summary, our data suggest that Blimp-1 plays a pivotal role in driving an effector program in ILC2s to promote type 2 immune response and limit worm infection, which is counterintuitive based on the heightened inflammatory state and altered surface phenotype of ILC2s in the *Nmur1*^Cre^*Prdm1*^fl/fl^ mice.

### Blimp-1 regulates effector cytokine production in ILC2s

**Figure S4.**
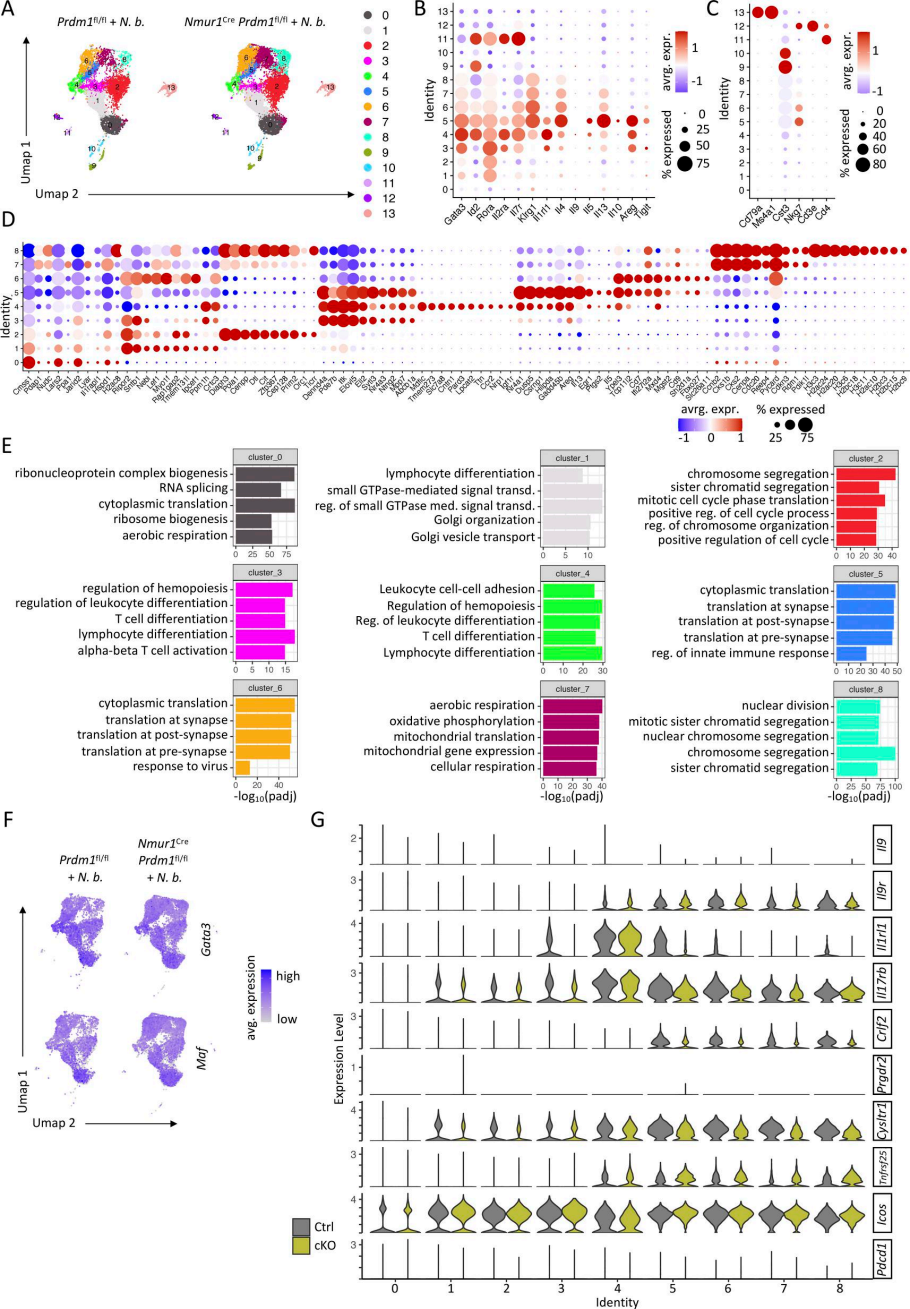
**scRNA-seq characterization of mLN ILC2s from** ***N. brasiliensis*** **infected** ***Prdm1*** **-deficient mice** **.** Related to Fig. 5. **(A)** UMAP of scRNA-seq data from mLN ILC2s of infected *Prdm1*^fl/fl^ and *Nmur1*^Cre^*Prdm1*^fl/fl^ mice before exclusion of non-ILC2 clusters (clusters 9–13). Cluster annotations are shown on the right. **(B)** Dot plots showing normalized expression levels of ILC2 marker genes in the clusters shown in A. **(C)** Dot plots showing normalized expression levels of lymphocyte marker genes in the clusters shown in A. **(D)** Dot plots showing normalized expression levels of the top 10 marker genes per cluster from Fig. 5 A. **(E)** GO analysis of the top upregulated genes per cluster from Fig. 5 A, showing the top five enriched pathways. **(F)** UMAPs of *Gata3* and *Maf* expression. **(G)** Violin plots showing normalized count distributions of different genes in different clusters in scRNA-seq of samples shown in A. *N*. *b*., *N*. *brasiliensis*. UMAP, uniform manifold approximation and projection.

**Figure 5.**
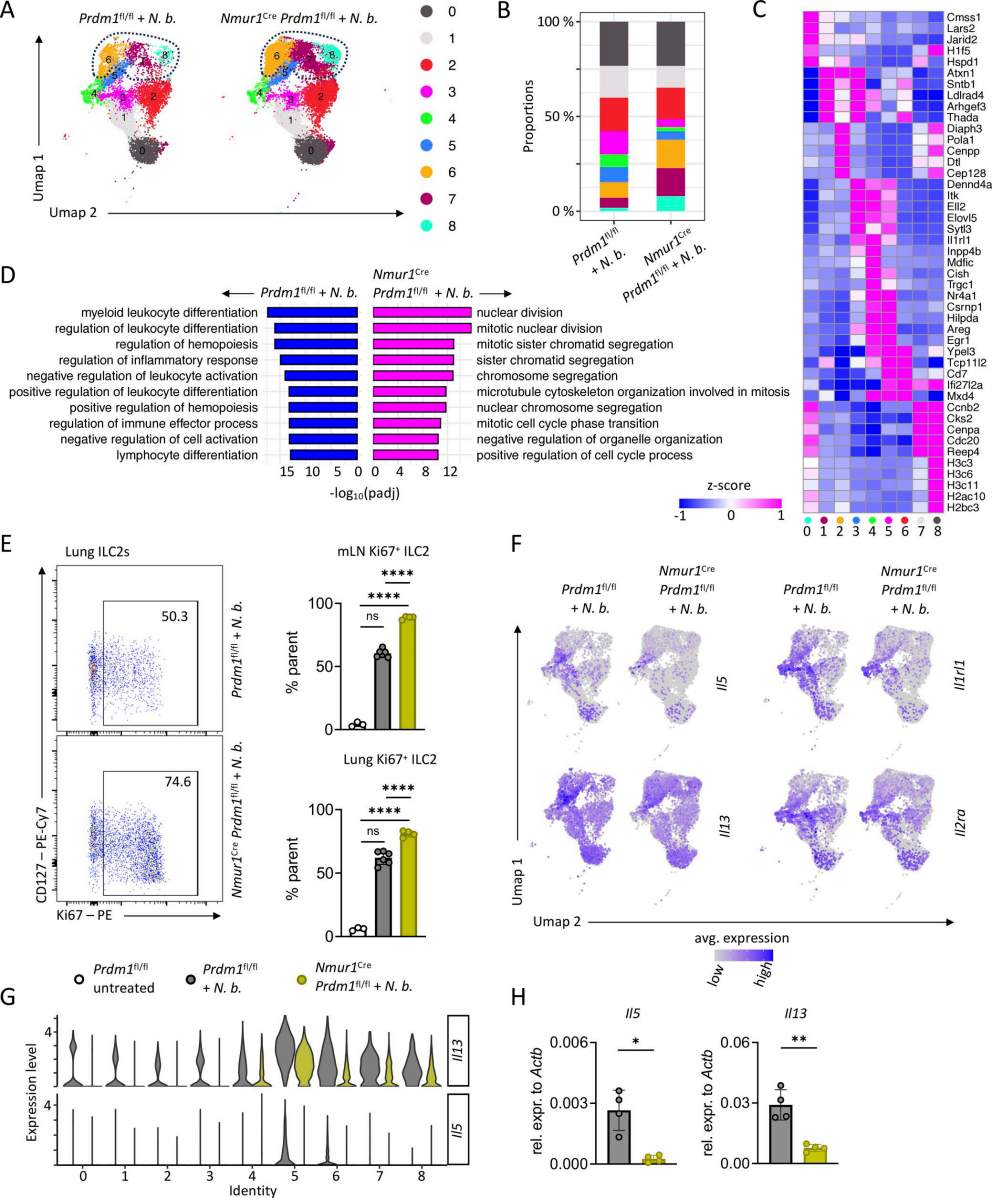
**Blimp-1–deficient ILC2s proliferate but exhibit reduced effector functions. (A)** UMAP of scRNA-seq of mLN ILC2s from infected Ctrl (*Prdm1*^fl/fl^) and cKO (*Nmur1*^Cre^*Prdm1*^fl/fl^) mice at day 7 of *N. brasiliensis* infection. Annotation of clusters on the right. **(B)** Frequencies of cells per cluster of samples shown in A. **(C)** Heatmap showing normalized count distributions of marker genes in different clusters in scRNA-seq of samples shown in A. **(D)** GO pathway analysis of DEGs between infected Ctrl and infected cKO mice in scRNA-seq of samples shown in A. **(E)** Flow cytometric plots and quantification of Ki67^+^ ILC2s in the mLN and lung in untreated and in infected Ctrl and infected cKO mice. Mice were infected with *N. brasiliensis* for 7 days. Data are representative of two independent experiments (*n* = 3–6 mice per group). **(F)** UMAPs of *Il5*, *Il13*, *Il1rl1*, and *Il2ra* expression. **(G)** Violin plots showing normalized count distributions of *Il5* and *Il13* in different clusters in scRNA-seq of samples shown in A. **(H)** Relative expression of *Il5* and *Il13* in lung ILC2s from infected Ctrl and cKO mice. Mice were analyzed 7 days after infection with *N. brasiliensis* (*n* = 4 mice per group). *N*. *b*., *N*. *brasiliensis*. Mean ± SD; one-way ANOVA. Student’s *t* test; *P < 0.05; **P < 0.01; ****P < 0.0001. UMAP, uniform manifold approximation and projection.

Despite being highly activated in Blimp-1 cKO mice, ILC2s failed to mount a protective immune response against the parasite *N. brasiliensis* (Fig. 4, A–F; and Fig. S3, A–E). To further dissect the role of Blimp-1 in ILC2s, we performed single-cell RNA-seq (scRNA-seq) of Blimp-1–sufficient and –deficient ILC2 populations in the mLN of *N. brasiliensis*–infected mice. We selected mLN ILC2s to obtain a reliable and representative population, which contains natural and iILC2s (Flamar et al., 2020). After excluding non-ILC2 clusters (Fig. S4, A–C), we obtained 9 ILC2 clusters (Fig. 5 A). A comparison between control ILC2s and ILC2s from cKO mice revealed an increase in clusters 6–8, which are outlined with dotted lines, after genetic ablation of *Prdm1* (Fig. 5, A and B). In these clusters, we observed upregulated genes related to histone remodeling (*H3c3*, *H2ac24*, and *H2ac20*) and cell cycle (*Ccnb2*, *Cks1b*, and *Cks2*), indicating the role of *Prdm1* in chromatin remodeling and upregulation of proliferation under inflammatory conditions (Fig. 5 C; and Fig. S4, D and E). Further, when investigating DEGs between control mice and cKO mice, GO pathway analysis showed upregulation of gene sets related to nuclear division, chromosome segregation, mitotic cell cycle phase transition, and similar processes, further supporting the idea that these clusters contain highly proliferative ILC2s (Fig. 5 D). Moreover, the upregulation of cell cycle and proliferation pathways in Blimp-1–deficient ILC2s is consistent with the higher number of ILC2s recovered from cKO mice and the increased percentage of Ki-67^+^ ILC2s (Fig. 4, A and B; and Fig. 5 E). In contrast, GO terms enriched in control ILC2s, such as “regulation of leukocyte differentiation,” “regulation of leukocyte activation,” and “regulation of immune effector process” (Fig. 5 D), suggest that Blimp-1–deficient ILC2s fail to acquire the immune effector programs necessary for effective worm resistance. Given the pivotal role of type 2 cytokines in controlling worm infections, we next analyzed the expression of these type 2 effector cytokines in ILC2s. Clusters 4 and 5 displayed the highest expression of *Il5* and *Il13*, together with a high expression of the *Il1rl1* and *Il2ra*. Consistent with previous results, we did not observe alteration in *Gata3*, *Maf, Il9r*, or *Icos* (Fig. 5, F and G; and Fig. S4, F and G). The cumulative evidence points toward type 2 cytokines and, in particular, IL-13, as an essential cytokine promoting the “weep and sweep reaction” in the epithelium for *N. brasiliensis* control (Cliffe et al., 2005; McKenzie et al., 1999). Indeed, *Il5* and *Il13* expression was decreased in Blimp-1–deficient ILC2s from the mLN (Fig. 5, F and G) and lung (Fig. 5 H) on day 7 after infection, which likely explains their susceptibility to *N. brasiliensis* infection. Taken together, these data provide evidence that Blimp-1 is required to drive a type 2 cytokine effector program in ILC2s, mediating worm expulsion.

### Blimp-1 drives type 2 effector cytokines in ILC2s and promotes eosinophilia during allergic lung inflammation

**Figure S5.**
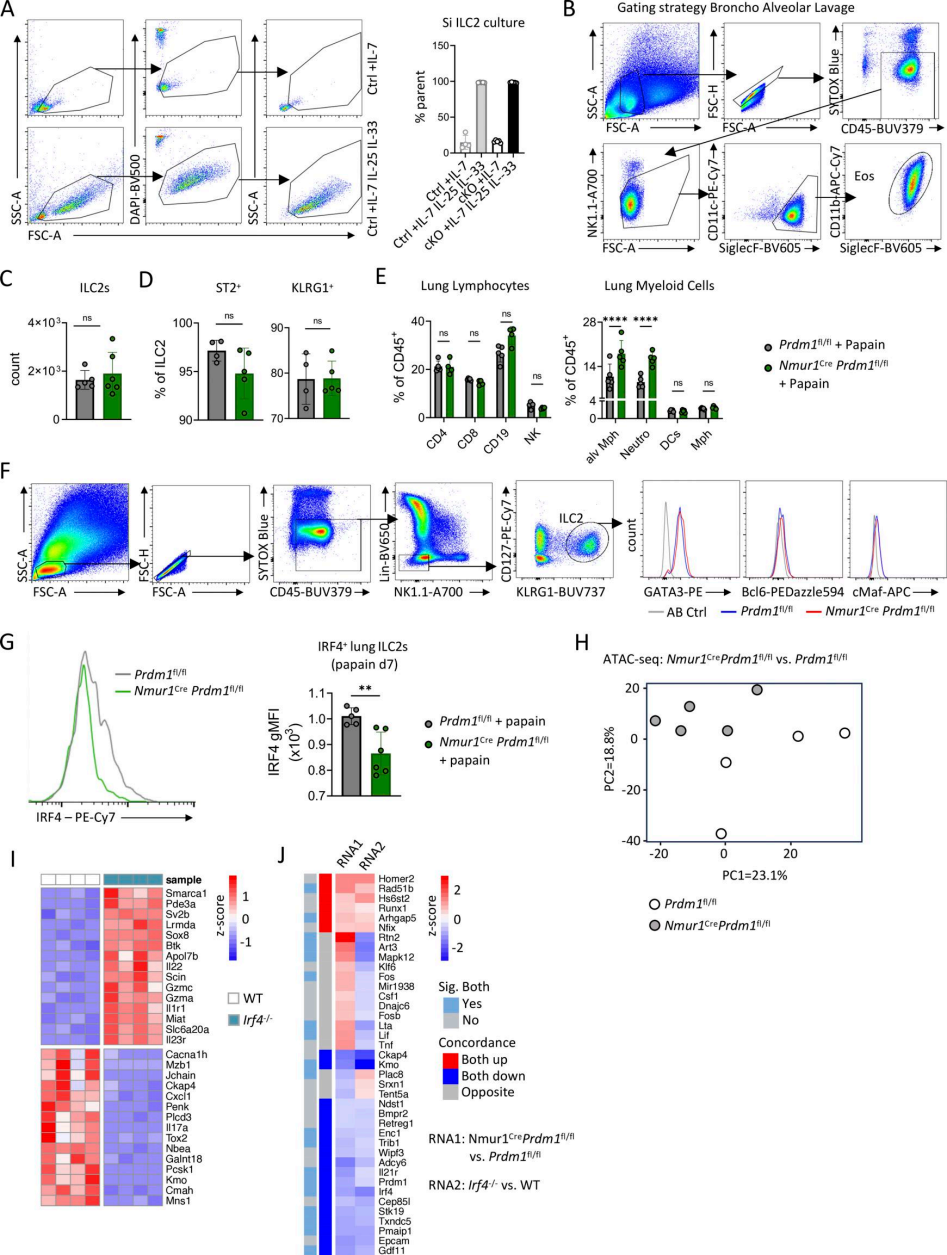
**Blimp1-deficient ILC2 effector functions and shared transcriptional programs with IRF4-deficient ILC2s** **.** Related to Fig. 6, 7, and 8. **(A)** Flow cytometry analysis and quantification of ILC2 activation of small intestinal ILC2s of *Prdm1*^fl/fl^ and *Nmur1*^Cre^*Prdm1*^fl/fl^ mice after 3 days stimulation with IL-7 or IL-7, IL-25, and IL-33 (*n* = 4–5 mice per group). **(B)** Gating strategy of bronchoalveolar lavage (BAL) eosinophils. **(C and D)** Quantification of flow cytometric analysis of ILC2 counts (C) and ST2^+^ and KLRG1^+^ ILC2s (D) in papain-treated Prdm*1*^fl/fl^ and *Nmur1*^Cre^*Prdm1*^fl/fl^ mice on day 7 after infection. **(E)** Quantification of flow cytometric analysis of lymphocytes and myeloid cells in papain-treated *Prdm1*^fl/fl^ and *Nmur1*^Cre^*Prdm1*^fl/fl^ mice on day 7 after infection. **(C–E)** Data are representative of two independent experiments (*n* = 4–6 mice per group). **(F)** Gating strategy of small intestinal ILC2s and the transcription factor staining shown in Fig. 7 F. **(G)** Flow cytometric analysis and quantification of IRF4^+^ ILC2s from papain treated *Prdm1*^fl/fl^ and *Nmur1*^Cre^*Prdm1*^fl/fl^ mice on day 7 after infection. Data are representative of two independent experiments (*n* = 4–6 mice per group). **(H)** PCA from bulk ATAC-seq of *Nmur1*^Cre^*Prdm1*^fl/fl^ vs. *Prdm1*^fl/fl^ mice; *n* = 4–5 mice per group. **(I)** Heatmap showing top 15 up- and downregulated genes from bulk RNA-seq of small intestinal ILC2s comparing naive *Irf4*^−/−^ and WT mice; *n* = 4–5 mice per group. **(J)** Heatmap showing concordance of DEGs between bulk RNA-seq datasets from *Nmur1*^Cre^*Prdm1*^fl/fl^ vs. *Prdm1*^fl/fl^ mice (RNA1) and *Irf4*^−/−^ vs. WT mice (RNA2). Mean ± SD; Student’s *t* test (C, D, and G) or one-way ANOVA (A and E); ns, nonsignificant; **P < 0.01; ****P < 0.0001.

**Figure 6.**
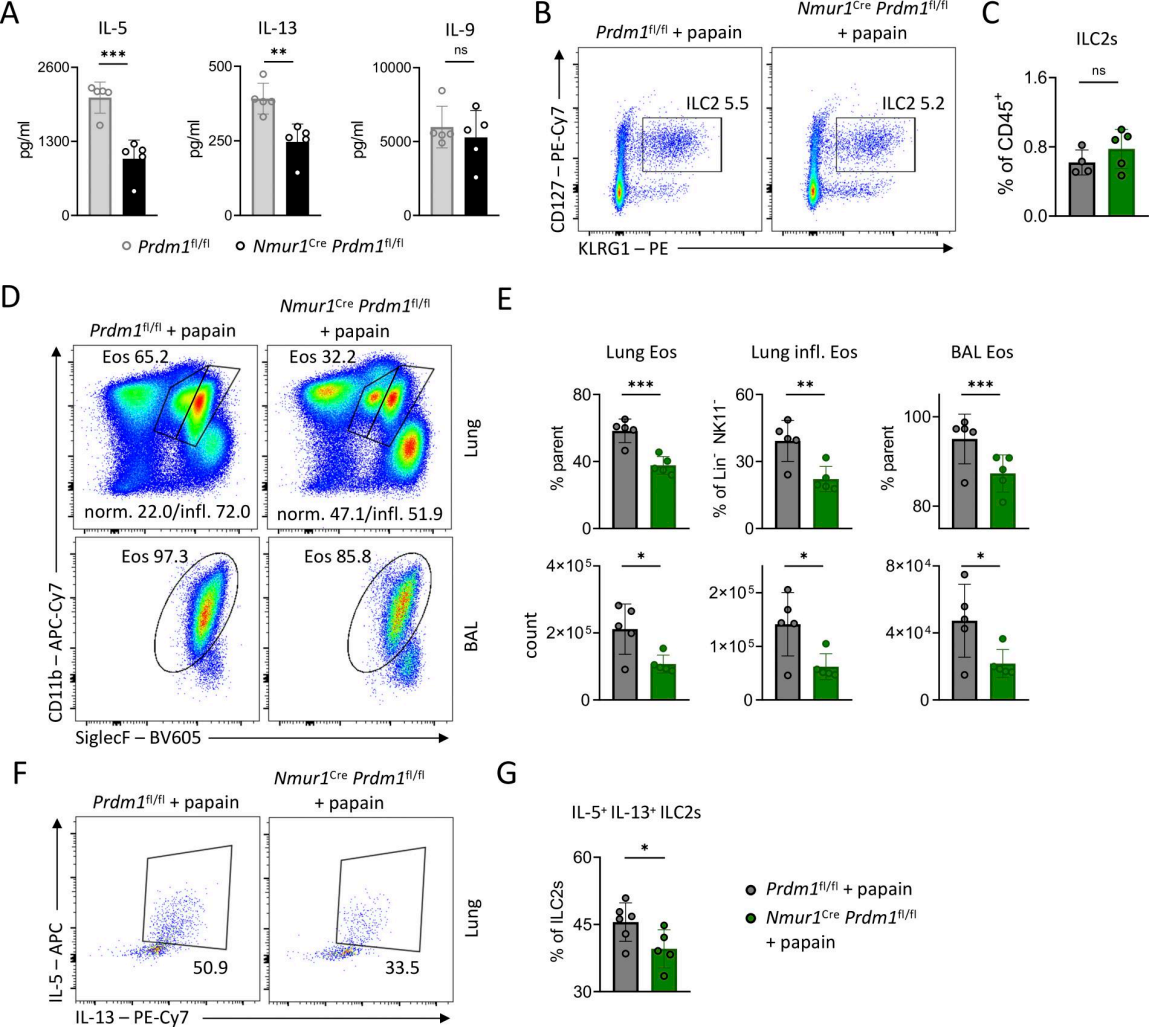
**Blimp-1–deficient ILC2s are poor type 2 cytokine producers *in vitro* and provoke less eosinophilia during allergic lung inflammation. (A)** Concentrations of the indicated cytokine as measured in the supernatant 3 days after stimulation of sort-purified small intestinal ILC2s of Ctrl (*Prdm1*^fl/fl^) and cKO (*Nmur1*^Cre^*Prdm1*^fl/fl^) mice with IL-7, IL-25, and IL-33 (*n* = 5 mice per group). **(B)** Representative flow cytometry plots showing lung ILC2s in papain-treated Ctrl and cKO mice. **(C)** Quantification of lung ILC2s (from B) in papain-treated Ctrl and cKO mice. **(D)** Representative flow cytometry plots showing lung and bronchoalveolar lavage (BAL) eosinophils in papain-treated Ctrl and cKO mice. **(E)** Quantification and counts of eosinophils (from D) in papain-treated Ctrl and cKO mice. **(F and G)** Flow cytometric analysis and quantification of IL-5– and IL-13–expressing lung ILC2s in papain-treated Ctrl and cKO mice. **(B–G)** Data are representative of two independent experiments; *n* = 4–5 mice per group. Student’s *t* test; *P < 0.05; **P < 0.01; ***P < 0.001.

Given the reduced IL-5 and IL-13 levels in Blimp-1–deficient ILC2s during *N. brasiliensis* infection, we hypothesized that Blimp-1 drives terminal differentiation and type 2 cytokine production in ILC2s. To test this hypothesis in a more reductionistic system, we performed *in vitro* cultures of sort-purified Blimp-1–sufficient and –deficient ILC2s and stimulated them with IL-7, IL-25, and IL-33. While activated ILC2s were comparable among both genotypes (Fig. S5 A), Blimp-1–deficient ILC2 secreted significantly less IL-5 and IL-13, similar to our results obtained in worm infection, but similar amounts of IL-9 protein as assessed by LEGENDplex multiplex assay in the cell culture supernatant (Fig. 6 A). Therefore, these data demonstrate that Blimp-1 is required for IL-5 and IL-13 production in ILC2s.

To investigate the *in vivo* relevance of Blimp-1 in a type 2 model unaffected by differences in pathogen burden, we challenged *Nmur1*^Cre^*Prdm1*^fl/fl^ and littermate controls intranasally with papain to induce allergic lung inflammation. Papain is not known to elicit iILC2s during allergic lung inflammation. While we did not detect notable differences in ILC2 numbers in this model, eosinophils and inflammatory eosinophils were significantly reduced in the lung and bronchoalveolar lavage of *Nmur1*^Cre^*Prdm1*^fl/fl^ mice compared with littermate controls (Fig. 6, B–E; and Fig. S5, B–E). Since eosinophils are regulated by ILC2s and IL-5, these data suggest that a reduction in ILC2 effector function is responsible for the decreased eosinophil numbers (Jarick et al., 2022; Jorssen et al., 2024; Kopf et al., 1996; Nussbaum et al., 2013; Topczewska et al., 2023). Indeed, Blimp-1–deficient lung ILC2s produced significantly less IL-5 and IL-13 compared with ILC2s from littermate controls during allergic lung inflammation (Fig. 6, F and G). Taken together, our data from *in vitro* and *in vivo* models support the view that Blimp-1–deficient ILC2s have a defective type 2 effector cytokine production.

### Blimp-1 deficiency results in reduced IRF4 expression

**Figure 7.**
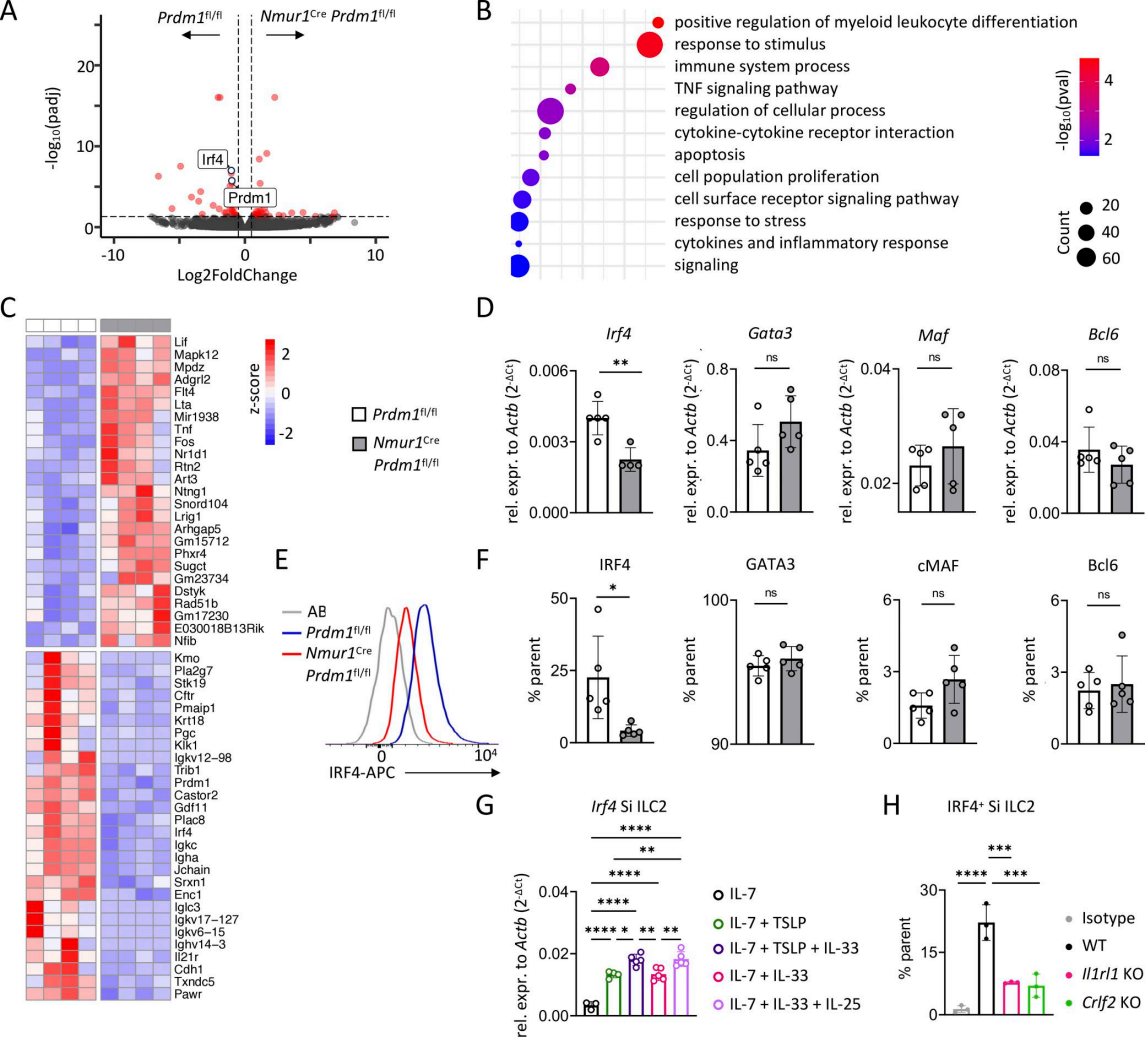
**IRF4 expression is dysregulated in Blimp-1–deficient ILC2s. (A)** Volcano plot showing differentially regulated genes in sort-purified small intestinal ILC2s from naive *Nmur1*^Cre^*Prdm1*^fl/fl^ (cKO) vs. *Prdm1*^fl/fl^ (Ctrl) mice. The genes *Prdm1* and *Irf4* are indicated in the plot. **(B)** GO pathway analysis of differentially regulated genes from bulk RNA-seq of small intestinal ILC2s comparing naive cKO and Ctrl mice. **(C)** Heatmap of the top differentially regulated genes from naive cKO and Ctrl mice. Genes of interest are indicated in red (A–C, *n* = 4 mice per group). **(D)** Relative expression of genes of interest in small intestinal ILC2s from naive cKO and Ctrl mice. **(E)** Histogram of flow cytometric analysis of IRF4 in intestinal ILC2s from naive cKO and Ctrl mice. **(F)** Quantification of E and of the transcription factors GATA3, cMaf and Bcl6 in intestinal ILC2s from naive cKO and Ctrl mice. **(G)** Relative expression of *Irf4* in sort-purified small intestinal (Si) ILC2s after *in vitro* stimulation with IL-7 alone, or with IL-7 or TSLP, or in combination with IL-33 and IL-7, IL-33, or in combination with IL-25. **(H)** Flow cytometric analysis of IRF4-expressing small intestinal (Si) ILC2s from WT, *Il33*^−/−^, and *Crlf2*^−/−^ mice. **(D–H)** Data are representative of two independent experiments; *n* = 3–5 mice per group. Mean ± SD; Student’s *t* test (D and F) or one-way ANOVA (G and H); ns, nonsignificant; *P < 0.05; **P < 0.01; ***P < 0.001; ****P < 0.0001.

To dissect Blimp-1–mediated gene regulation in ILC2s under homeostatic conditions, we performed bulk RNA-seq on sort-purified intestinal ILC2s from *Nmur1*^Cre^*Prdm1*^fl/fl^ and littermate controls at steady state. Blimp-1 deficiency resulted in altered gene expression (Fig. 7 A), with enriched pathways related to “response to stimulus,” “cytokine-cytokine receptor interaction,” or “cytokines and inflammatory response” (Fig. 7 B). These findings highlight the role of Blimp-1 in regulating a network involved in cytokine responsiveness. As expected, *Prdm1* was downregulated in *Nmur1*^Cre^*Prdm1*^fl/fl^ mice (Fig. 7 A). We did not find differences in the expression of *Gata3*, *Bcl6*, or c*Maf*, which have previously been shown to interact with Blimp-1 in other cell types (He et al., 2020; Howard et al., 2021; Shaffer et al., 2002). However, the transcription factor *Irf4* was significantly downregulated in Blimp-1–deficient ILC2s to a similar degree as *Prdm1* (Fig. 7, A, C, and D). Moreover, a subset of signaling-related genes (*Lif, Tnf, Fos, Lrig1, Nfib, Mapk12,* and *Flt4*) was increased (Fig. 7, B and C). This downregulation of IRF4 was confirmed at both the mRNA and protein levels, establishing IRF4 as a target of Blimp-1 in ILC2s (Fig. 7, D–F; and Fig. S5 F). To investigate whether alarmins regulate IRF4, we stimulated ILC2s *in vitro* with a combination of cytokines, including TSLP, IL-25, and IL-33. Both TSLP and IL-33 stimulation alone induced *Irf4* expression in ILC2s, while co-stimulation with TSLP and IL-33 or IL-33 and IL-25 led to a further increase in *Irf4* expression (Fig. 7 G). In our allergic lung inflammation model, lung ILC2s lacking *Prdm1* also exhibited reduced IRF4 expression, indicating that Blimp-1 is required to maintain IRF4 levels *in vivo* (Fig. S5 G). Moreover, to test whether the loss of alarmin signaling results in reduced IRF4 protein levels *in vivo*, we analyzed IRF4 expression in ILC2s from *Il33*^−/−^ and *Crlf2*^−/−^ mice by flow cytometry. Consistent with our *in vitro* findings, IRF4 was diminished in ILC2s from TSLP- and IL-33–deficient mice (Fig. 7 H), indicating that these two alarmins regulate IRF4. Since IRF4 has been previously linked to *N. brasiliensis* resistance and ILC2 activation, the reduced IRF4 expression could at least partially explain the impaired anti-helminth response in Blimp-1 cKO mice. These findings further position IRF4 as a downstream transcription factor of alarmin signaling, providing a mechanistic link between Blimp-1 and ILC2 effector function (Cretney et al., 2011; Honma et al., 2008; Mohapatra et al., 2016). Therefore, our data uncover a pivotal signaling pathway downstream of the alarmin IL-33, which involves Blimp-1 and IRF4 for ILC2 activation and protective type 2 immune response in anti-worm immunity, but may contribute to pathogenesis of allergic diseases.

### Blimp-1 and IRF4 form a co-regulatory module integrating IL-33–dependent transcriptional programs to control ILC2 effector function

**Figure 8.**
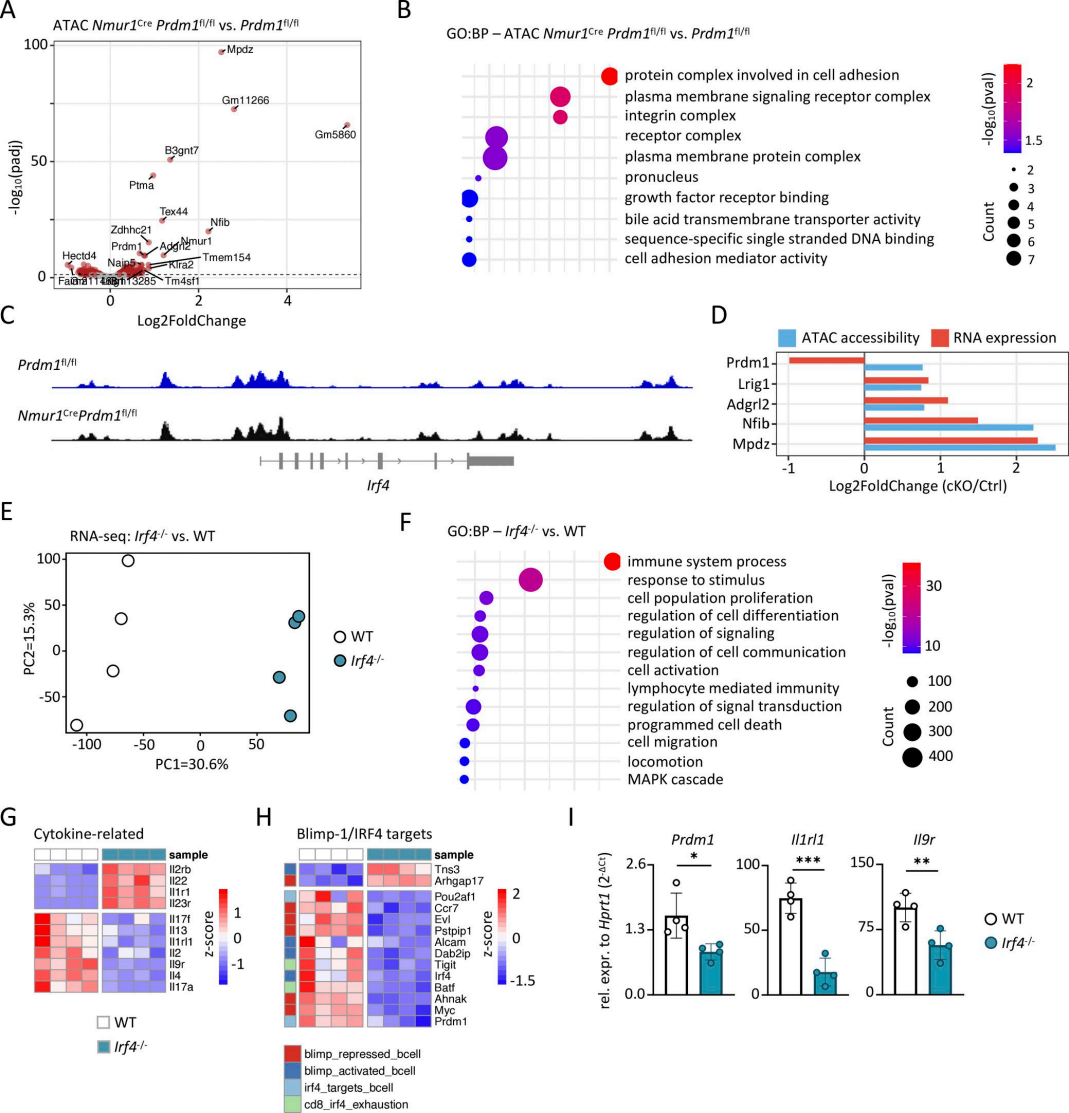
**The IL-33–Blimp-1–IRF4–ST2 axis regulates ILC2 effector functions. (A)** Volcano plot of ATAC-seq peaks showing differentially accessible chromatin regions in sort-purified small intestinal ILC2s from naive cKO (*Nmur1*^Cre^*Prdm1*^fl/fl^) vs. Ctrl (*Prdm1*^fl/fl^) mice. **(B)** GO pathway analysis of differentially accessible chromatin regions identified by ATAC-seq in small intestinal ILC2s from naive cKO vs. Ctrl mice. **(C)** IGV genome browser tracks showing ATAC-seq signal at the *Irf4* locus in sort-purified small intestinal ILC2s from Ctrl and cKO mice. **(D)** Bar plot comparing DESeq2 results from ATAC and bulk RNA-seq from small intestinal ILC2s in cKO vs. Ctrl mice. **(E)** PCA of bulk RNA-seq of small intestinal ILC2s from *Irf4*^−/−^ vs. WT mice. **(F)** GO pathway analysis of differentially regulated genes from bulk RNA-seq of small intestinal ILC2s comparing naive *Irf4*^−/−^ and WT mice. **(G and H)** Heatmap showing cytokine-related genes (G) and Blimp-1/IRF4 targets from literature (Cretney et al., 2011; Kwon et al., 2009; Man et al., 2017; Minnich et al., 2016; Ochiai et al., 2013; Tellier et al., 2016; Vasanthakumar et al., 2015) (H) from bulk RNA-seq of small intestinal ILC2s comparing naive *Irf4*^−/−^ and WT mice. **(I)** Relative expression of *Prdm1*, *Il1rl1*, and *Il9r* from small intestinal ILC2s from *Irf4*^−/−^ and WT mice (*n* = 4–5 mice per group). Mean ± SD; Student’s *t* test; *P < 0.05; **P < 0.01; ***P < 0.001.

To dissect how Blimp-1 shapes ILC2 effector programs, we first profiled chromatin accessibility in sort-purified small intestinal ILC2s from naive *Nmur1*^Cre^*Prdm1*^fl/fl^ and *Prdm1*^fl/fl^ control mice using the assay for transposase-accessible chromatin sequencing (ATAC-seq). Differential peak analysis revealed a distinct chromatin landscape in Blimp-1–deficient ILC2s (Fig. S5 H). Among the most significantly altered regions were peaks associated with *Nfib*, *Mpdz*, and *Adgrl2* (Fig. 8 A). GO analysis of the differentially accessible regions showed enrichment for pathways related to protein complexes involved in cell adhesion, plasma membrane signaling receptor complexes, integral membrane receptor complexes, pronucleus-associated complexes, and growth factor receptor binding (Fig. 8 B). These altered pathways point to altered accessibility at loci involved in receptor signaling and cell–cell communication, consistent with the impaired responsiveness of Blimp-1 cKO mice to inflammatory cues. However, analysis of chromatin accessibility at the *Irf4* locus showed no differences between control and *Prdm1*-deficient ILC2s (Fig. 8 C), indicating that Blimp-1 does not directly regulate *Irf4* via local chromatin remodeling under steady state conditions. To relate chromatin accessibility to transcriptional output, we compared the ATAC-seq and RNA-seq datasets from *Nmur1*^Cre^*Prdm1*^fl/fl^ vs. *Prdm1*^fl/fl^ ILC2s from the small intestine. While several loci showed coordinated decreases in accessibility and gene expression, including *Lrig1*, *Adgrl2*, *Nfib*, and *Mpdz*, *Prdm1* itself displayed reduced transcription, while we found increased chromatin accessibility at its locus (Fig. 8 D), suggesting a potential self-regulatory feedback loop.

To determine whether IRF4 participates in the same regulatory module as Blimp-1, we performed bulk RNA-seq on sort-purified small intestinal ILC2s from naive *Irf4*^−/−^ and WT controls (Fig. 8, E–H; and Fig. S5 I). Similar to *Prdm1*-deficient ILC2s, GO analysis revealed enrichment of pathways associated with immune system processes, cytokine signaling, and regulation of stimulus responses (Fig. 8 F), indicating that IRF4 and Blimp-1 converge on overlapping effector programs. However, the transcriptional consequences of IRF4 loss were even broader than in Blimp-1–deficient ILC2s. Analysis of the most strongly regulated genes revealed that several of the top upregulated transcripts in *Irf4*^−/−^ ILC2s belonged to the type-17 pathway, including *Il22*, *Il22b*, and *Il23r* (Fig. S5 I). This enrichment prompted us to further examine cytokine-related genes in a targeted manner. Indeed, a comparison of cytokine and cytokine-receptor genes demonstrated downregulation of type 2–associated transcripts, including *Il4*, *Il13*, *Il9r*, *Il7r*, and the IL-33 receptor *Il1rl1*, whereas type 17 markers were elevated (Fig. 8 G). These data suggest that IRF4 is required to maintain type 2 identity while actively suppressing type 17–associated gene programs in intestinal ILC2s.

To further investigate whether IRF4 regulates a broader effector module beyond cytokines, we next examined a curated set of genes identified in the literature as Blimp-1 or IRF4 targets in plasma cells (Minnich et al., 2016; Ochiai et al., 2013; Tellier et al., 2016), CD4^+^ T cells (Kwon et al., 2009), regulatory T cells (Cretney et al., 2011; Vasanthakumar et al., 2015), or exhausted CD8^+^ T cells (Man et al., 2017) (Fig. 8 H). Strikingly, among these genes, *Prdm1* was reduced in *Irf4*-deficient ILC2s. This reduction in *Prdm1* expression indicates that IRF4 might contribute to maintaining Blimp-1 levels in ILC2s and is consistent with a role for IRF4 as a co-regulator of the Blimp-1–associated transcriptional programs. Other canonical target genes dysregulated in *Irf4*^−/−^ ILC2s include *Batf*, *Tigit*, *Pou2af1*, and *Myc*. Validation using qPCR confirmed the reduction of *Prdm1*, *Il1rl1*, and *Il9r* in *Irf4*–deficient ILC2s (Fig. 8 I), verifying the RNA-seq findings. Finally, direct comparison of the *Prdm1*- and *Irf4*-deficient transcriptional signatures revealed overlap of regulated genes (Fig. S5 J), supporting the view that these transcription factors might operate within a shared regulatory axis.

Together, these data show that IRF4 regulates the type-2 activation program, including expression of the IL-33 receptor ST2 and the downstream effector regulator Blimp-1. Our data indicate that IRF4-deficient ILC2s display a reduced potential to produce type 2 cytokine potential, with increased type 17–associated transcripts. These findings identify the IL-33–IRF4–Blimp-1 axis as a central regulatory module that controls ILC2 effector function while suppressing ILC3-related effector molecules.

## Discussion

Alarmins are potent stimulators of ILC2s and type 2 inflammation, highlighting them as therapeutic targets for allergic diseases in humans (Menzies-Gow et al., 2021; Stanbery et al., 2022). Type 2 effector cytokines are in the focus for neutralization by biologicals for treatment of asthma also (Wechsler et al., 2021). However, how distinct alarmin signals are differentially perceived by ILC2s and integrated in the downstream signaling networks that control type 2 effector response is an outstanding question in the field today. While alarmins are dispensable for ILC2 development, they are essential for ILC2 activation and cytokine production (Ricardo-Gonzalez et al., 2018; Topczewska et al., 2023). IL-25 is predominantly linked to the differentiation of iILC2, whereas IL-33 can promote the phenotype as well (Flamar et al., 2020; Huang et al., 2015). Therefore, IL-25 plays a major role in gut responses and worm infections, where small intestinal ILC2s reside, characterized by low ST2 expression. Nevertheless, our study highlights IL-33 as an essential alarmin signaling pathway controlling effector cytokine production and cell cycle progression in ILC2s.

Our sequencing data obtained in small intestinal ILC2s revealed that *Prdm1* is mainly regulated by the IL-33 receptor signaling at steady state, but not by the IL-25 receptor. In contrast, following worm infection, *Prdm1* was downregulated in ILC2s from IL-33– and IL-25R–deficient mice, correlating with the susceptibility of both strains to *N. brasiliensis* infection (Hung et al., 2013; Neill et al., 2010). These findings are further consistent with literature showing that IL-25 and IL-33 jointly drive ILC2 activation and promote iILC2 differentiation. Therefore, deficiency in either pathway compromises worm immunity (Flamar et al., 2020; Neill et al., 2010; Topczewska et al., 2023). These data support a model in which Blimp-1 integrates IL-25 and IL-33 inputs during infection, while IL-33 remains the predominant regulator at steady state.

Since Blimp-1–deficient ILC2s maintained proliferative capacity but produced limited amounts of type 2 effector cytokines, Blimp-1 could be responsible for modulating these functions in ILC2s. However, whether this reflects the predominant action of IL-33 or joint IL-25 and IL-33 signals remains to be further investigated.

As also evidenced by our sequencing data set, iILC2 are capable of migrating from the intestine to other organs, such as the lungs, where they contribute to allergic lung inflammation. This migration process has been linked to sphingosine phosphate receptors, ICOS, and Tph1 signaling in ILC2, and several of these molecules were identified in our sequencing data (Burrows et al., 2025; Flamar et al., 2020; Huang et al., 2018). We detected an unprecedented phenotype in Blimp-1–deficient mice, where highly proliferative iILC2 infiltrated LNs and lungs, which were still ineffective in fighting worm infection due to defective type 2 cytokine production, namely of the IL-5 and IL-13. IL-13 plays a critical role for tuft and goblet cell hyperplasia, processes essential for worm expulsion (Herbert et al., 2009; McKenzie et al., 1999; von Moltke et al., 2016). Consistent with this, ILC2s represent the dominant source of IL-13 during the first 7–10 days of *N. brasiliensis* infection, and ILC2-derived IL-13 is required to initiate epithelial hyperplasia. After the early phase, Th2 cells become the main IL-13 producers, contributing to maintenance rather than initiation of expulsion, as shown previously using cytokine reporter mice (Zaiss et al., 2024). This division is further supported by work by Gurram et al. (2023), which demonstrates that Th2-deficient mice can eventually expel *N. brasiliensis*, whereas ILC2-deficient and Th2-deficient mice were infected even at day 200 after infection, showing that each population contributes nonredundantly to efficient parasite clearance. In Blimp-1–deficient ILC2s, the diminished production of IL-13 during this critical initiation phase likely prevents the epithelial differentiation and hyperplasia necessary for effective worm clearance. The residual IL-13 detected in these mice is therefore insufficient to restore protective immunity. Based on our dataset and previous studies (Neill et al., 2010; Oeser et al., 2015; Varela et al., 2022), this remaining IL-13 is most plausibly derived from early Th2 cells, rather than from ILC2s themselves. However, the timing and quantity of IL-13 produced by these alternative populations appear inadequate to compensate for the lack of robust ILC2-derived cytokine production.

We further observed that Blimp-1 deficiency impaired IL-5 and IL-13, but not IL-9 production of *in vitro* stimulated ILC2s. Interestingly, IL-4, IL-5, and IL-13 are regulated by the Th2 locus control region localized in the RAD50 gene (Lee et al., 2003). Although we did not observe changes at this locus in *Prdm1*-deficient ILC2s under steady state conditions, it might be regulated under inflammatory conditions, as this could explain the selective regulation of IL-5 and IL-13 but not IL-9 by Blimp-1 in ILC2s.

Transcriptional regulation of ILCs has been mainly studied in development, which helped in the initial years of the field to delineate ILC lineages and progenitor populations. Key transcription factors such as GATA-3, ID2, and ROR-α are required for lineage commitment in ILC progenitors or for ILC2 development after lineage commitment, resulting in the absence of ILC2s in gene-deficient mice (Furusawa et al., 2013; Halim et al., 2012; Hoyler et al., 2012; Klein Wolterink et al., 2013; Moro et al., 2010; Wong et al., 2012; Yu et al., 2015). Consequently, the role of transcription factors after ILC2 development remains largely unexplored, and the signaling networks mediating ILC2 activation are yet to be fully elucidated. In this regard, the transcription factor BATF is required for the differentiation of iILC2 during worm infection (Miller et al., 2020), and our sequencing data showed a reduction of *Batf* in IL-25R–deficient and IRF4-deficient ILC2s. Based on these findings and given that BATF can bind to the *Prdm1* promoter, BATF could be part of the signaling network controlled by Blimp-1–IRF4 axis (Fu et al., 2017; Miller et al., 2020). However, how BATF integrates into the regulatory network around Blimp-1 and IRF4 in ILC2s remains to be investigated. In addition, iILC2s, which are predominately regulated by IL-25, do not differentiate in *Batf*^−/−^ mice. In contrast, Blimp-1 is mainly regulated by IL-33, and although iILC2s are well represented in Blimp-1–deficient mice, they fail to produce cytokines needed to control worm infection. These data suggest that proliferation and differentiation into iILC2, on the one hand, and effector cytokine production, on the other, are functioning independently at the cellular level and that underlying molecular modules are regulated by Blimp-1, BATF, or IRF4, including overlapping and distinct gene regulation and context-dependent modulation. Interestingly, Blimp-1 has been shown to be required for the differentiation of several immune cells, including but not limited to Th2 cells (He et al., 2020). In Th2 cells, Blimp-1 promotes the differentiation through BCL6 and increases GATA-3 expression downstream of the IL-10 receptor. Given the shared requirement for type 2 cytokine production in both, Th2 and ILC2s, Blimp-1 inhibitors could be considered as a target for allergy treatment to interfere with innate and adaptive lymphocytes, simultaneously (He et al., 2020). However, in certain cell types, such as regulatory T cells and lung ILC2s, Blimp-1 has also been linked to IL-10 secretion (Cretney et al., 2011; Howard et al., 2021). We did not find evidence that Blimp-1 mediates similar effects in ILC2s, but could link it to IRF4, a transcription factor known to regulate the differentiation of Th2, Th9, and Th17 cells. In T cells, IRF4 has been shown to directly control cytokine production by binding to the AP-1/IRF motif together with BATF, thereby regulating cytokine expression in effector T cell subsets (Li et al., 2012). On the other hand, IRF4 has been found to sustain a feed-forward loop in cancer cells, promoting the NF-κB signaling pathway, which is downstream of both, the IL-25 and IL-33 signaling (Boddicker et al., 2015; Wong et al., 2020).

Our integrative ATAC-seq and RNA-seq analyses uncovered that IL-33 drives an IRF4-Blimp-1 transcriptional module that is essential for ST2 (*Il1rl1*) expression and type-2 effector differentiation. *Prdm1* exhibited reduced transcription yet increased chromatin accessibility in Blimp-1–deficient ILC2s, consistent with a potential autoregulatory feedback loop. In parallel, both *Prdm1* and *Il1rl1* were downregulated in *Irf4*^−/−^ ILC2s, indicating that IRF4 regulates Blimp-1 and maintains IL-33 responsiveness. This regulatory pathway resembles circuits in regulatory T cells, where BATF and IRF4 cooperate to induce *Il1rl1* and *Pparg* expression, enabling IL-33 responsiveness and tissue adaptation (Vasanthakumar et al., 2015).

Importantly, IRF4 deficiency resulted in a stronger transcriptional dysregulation than Blimp-1 deficiency. *Irf4*^−/−^ ILC2s showed a profound shift toward type 17–associated gene expression, including *Il22*, *Il22b*, and *Il23r*, while downregulating classical type 2 cytokine genes (*Il4* and *Il13*) and survival signals (*Il7r*). Notably, the expression of *Il9r* and the IL-33 receptor *Il1rl1* was also reduced in IRF4-deficient, but not in Blimp-1–deficient, ILC2s. These findings align with prior work, where Mohapatra et al. showed that IL-33 and TSLP synergistically induce an IRF4–IL-9 module in lung ILC2s, in which IRF4 drives IL-9 production and IL-9 amplifies IL-5 and IL-13 responses (Mohapatra et al., 2016). The reduction of *Il9r* in *Irf4*^−/−^ ILC2s extends the IRF4–IL-9 axis to intestinal ILC2s and indicates that IRF4 regulates not only IL-9 production but also IL-9 responsiveness. Reduced IL-9R expression might be an additional explanation for the impaired type 2 effector function in the absence of IRF4, as IL-9 signaling promotes ILC2 survival, proliferation, and cytokine production. Together, these findings support a model in which IRF4 operates upstream of Blimp-1 to coordinate alarmin-driven activation by IL-33, sustain the IL-9–dependent feed-forward circuit required for robust type 2 immunity, and simultaneously restrain alternative effector programs. In this way, IRF4 acts as a lineage-stabilizing transcription factor that maintains ILC2 identity while preventing diversion toward type 17–associated gene expression.

Moreover, our findings help explain why both *Irf4*- and *Prdm1*-deficient mice show similar defects during *N. brasiliensis* infection. In both cases, ILC2s fail to produce enough type 2 cytokines, leading to poor worm clearance. Earlier studies showed that IRF4 was required for resistance to *N. brasiliensis* infection (Honma et al., 2008), but these experiments were done before ILC2s were discovered (Moro et al., 2010; Neill et al., 2010; Price et al., 2010), leaving the responsible cell type unclear. Later work demonstrated that *Irf4*^−/−^ mice cannot mount proper ILC2 responses during infection (Mohapatra et al., 2016). By showing that Blimp-1 expression in ILC2s depends on IRF4, our study connects these findings and provides a mechanistic explanation. However, future studies are necessary to investigate and dissect how IRF4 precisely links to the Blimp-1 pathway on a molecular level.

Our data indicating that IRF4 regulates Blimp-1 and that both transcription factors co-govern a shared set of effector genes, which are well-established in adaptive lymphocytes. In B cells, IRF4 directly initiates plasma cell differentiation by activating *Prdm1* (Kwon et al., 2009; Ochiai et al., 2013), while Blimp-1 enforces terminal differentiation by repressing B cell identity genes such as *Pax5, Bcl6, SpiB,* and *Ciita* and activating secretory programs, including *Ell2, Mzb1,* and the immunoglobulin loci (Minnich et al., 2016; Tellier et al., 2016). Importantly, Minnich et al. demonstrated extensive cross-regulation between IRF4 and Blimp-1 and identified many of the genes that we now find dysregulated in *Irf4*-deficient ILC2s. A similar cooperation was found in regulatory T cells, where IRF4 is essential for effector regulatory T cell differentiation and for induction of Blimp-1, which controls IL-10 production and tissue residency (Cretney et al., 2011). Likewise, as already mentioned, IL-33–responsive visceral adipose tissue regulatory T cells require IRF4, BATF, and Blimp-1 for expression of ST2 (*Il1rl1*) and PPARγ (Vasanthakumar et al., 2015), highlighting a conserved role for the IRF4–Blimp-1 module in alarmin-dependent effector specialization. Finally, in chronically stimulated CD8^+^ T cells, IRF4 is required to establish the exhaustion program, driving inhibitory receptor expression (*Havcr2*, *Tigit*, and *Pdcd1*) and suppressing memory-associated genes, such as *Tcf7, Ccr7,* and *Il7r* (Man et al., 2017).

In summary, our study identifies a central IL-33–Blimp-1–IRF4 transcriptional axis that governs ILC2 effector function, maintains type-2 identity, and prevents alternative cytokine programs. This regulatory module explains shared features in IRF4- and Blimp-1–deficient ILC2s, links alarmin signaling to transcriptional control, and provides a mechanistic foundation for therapeutic targeting of type 2 inflammation.

## Materials and methods

### Mouse strains

C57BL/6 mice (*Mus musculus*) were purchased from Janvier. *Nmur1*^Cre^ (Jarick et al., 2022; Tsou et al., 2022), *Prdm1*^fl/fl^ (Shapiro-Shelef et al., 2003), *Zc3h12c*^fl/fl^ (provided by The European conditional mouse mutagenesis program [EUCOMM]), *Il17rb*^−/−^ (Neill et al., 2010), *Il33*^−/−^ (Oboki et al., 2010), *Il1rl1*^−/−^ (Neill et al., 2010), and *Crlf2*^*−/−*^ (Al-Shami et al., 2004) on a C57BL/6 background were bred locally at Charité animal facility. Blimp-1–YFP (Fooksman et al., 2014) mice were purchased from the Jackson Laboratory (B6.Cg-Tg(Prdm1-EYFP)1Mnz/J, RRID: IMSR_JAX:008828). *Irf4*^*−/−*^ mice (Mittrucker et al., 1997) were kindly provided by Hans-Willi Mittrücker and Blimp-1–eGFP mice (Kallies et al., 2004) by Anja E. Hauser. Sex- and age-matched male and female animals, usually aged 7–14 wk, were used for experiments. All animal experiments were approved and are in accordance with the local animal care committees (Lageso Berlin under application number G0158/19, T-CH-0023/22, and the Institutional Animal Care and Use Committee at Weill Cornell Medicine).

### Cell isolation

Small intestine was removed, cleaned from remaining fat tissue, and washed in ice-cold PBS. Peyer’s patches were eliminated, and small intestine was opened longitudinally and washed in ice-cold PBS. Dissociation of epithelial cells was performed by incubation on a shaker at 37°C in HBSS (Sigma-Aldrich) containing 10 mM Hepes (Gibco) and 5 mM EDTA (Roboklon) two times for 15 min. After each step, samples were vortexed, and the epithelial fraction was discarded. Afterward, remaining tissue was chopped into small pieces, and enzymatic digestion was performed using dispase (0.5 U/ml; Corning), collagenase D (0.5 mg/ml; Roche), and DNaseI (100 μg/ml; Sigma-Aldrich). Leukocytes were further enriched by Percoll gradient centrifugation (GE Healthcare). Lungs were chopped and incubated in the enzyme cocktail described above for 40 min on a shaker at 37°C. The remaining tissues were mashed with a syringe plunger, and single-cell suspensions were filtered through a 70-μm cell strainer. Leukocytes were then further enriched by Percoll gradient centrifugation. mLNs were chopped and incubated in RPMI 1640 medium (Gibco) supplemented with 1% BSA (Sigma-Aldrich), collagenase II (1 mg/ml; Sigma-Aldrich), and DNaseI (100 μg/ml) for 20 min on a shaker at 37°C. Afterward, cells were dissociated using a Pasteur pipette and filtered through a 70-μm cell strainer. Epididymal white adipose tissue was removed and incubated in the same digestion buffer for 45 min on a shaker at 37°C. After incubation, cells were dissociated using a Pasteur pipette, filtered through a 70-μm cell strainer and spun down, and the adipocyte layer was aspirated. For isolation of bone marrow cells, femur and tibia bone were crushed with a pestle and rinsed, and cells were filtered through a 70-μm cell strainer. Red cell lysis was performed in ACK lysis buffer for 3 min.

### Flow cytometry and cell sorting

Dead cells were routinely excluded with Fixable Aqua Dead Cell Stain or SYTOX Blue Dead Cell Stain (Thermo Fisher Scientific). Single-cell suspensions were incubated on ice with anti-CD16/CD32 antibody and the following conjugated antibodies in PBS (Ca^2+^ and Mg^2+^-free, Sigma-Aldrich). If indicated, lineage-positive cells were excluded by staining for CD3ε (145-2C11 or 500A2 or 17A2), CD5 (53–7.3), CD19 (1D3 or 6D5), FcεRI (Mar-1), Ly6G (1A8), CD4 (GK1.5), and B220 (RA3-6B2). For surface staining, the following antibodies were used: CD101 (REA301, Miltenyi), c-Kit (2B8), CD11b (M1/70), CD11c (N418), IL-25R (Munc33), CD127 (A7R34), CD19 (1D3), CD4 (GK1.5 and RM4-5), CD45 (30-F11) or CD45.2 (104), CD90 (30H12), F4/80 (BM8), KLRG1 (2F1 or MAFA), NK1.1 (PK136), PD-1 (29F.1A12), Sca-1 (D7), Siglec F (E50-2440), ST2 (RMST2-33), and TSLPR (22H9). Rat IgG2bκ and IgG1 Iso were used as controls. Before staining Ki-67 (B56), IRF4 (3E4), GATA3 (L50-823 and TWAJ), Bcl6 (7D1), cMaf (sym0F1), intracellularly, the cells were fixed by using the Foxp3 transcription factor buffer set (Thermo Fisher Scientific). All antibodies used in flow cytometry were purchased from eBioscience, BioLegend, or BD Biosciences if not otherwise indicated. All flow cytometry experiments were acquired using a custom configuration Fortessa flow cytometer and the FACS Diva software (BD Biosciences) and were analyzed with FlowJo version 9.9.3 or version 10.6.2 software (TreeStar) or sort-purified by using a custom configuration FACSAria cell sorter (BD Biosciences).

### Quantitative real-time PCR

Sorted cells were homogenized in Trizol (Thermo Fisher Scientific) and stored at −80°C. RNA was extracted with chloroform, and RNA concentration was determined using a NanoDrop 2000 spectrophotometer (Thermo Fisher Scientific). Reverse transcription of total RNA was performed using the High Capacity cDNA Reverse Transcription kit according to the protocol provided by the manufacturer (Thermo Fisher Scientific). Reaction was detected on a QuantStudio 5 Real-Time PCR (Thermo Fisher Scientific) using Taqman Gene Expression Assay (Applied Biosystems) with *Prdm1* (Mm00476128_m1), *Zc3h12c* (Mm01177355_m1), or SYBR Green Master Mix using the following primers: *Prdm1* (forward: 5′-TCC​CGA​GGT​TTC​TGG​CTA​TTG-3′, reverse: 5′-CCA​GAA​TGC​AAT​CGA​AGG​TGG-3′), *Irf4* (forward: 5′-GGC​CCA​ACA​AGC​TAG​AAA​GAG-3′, reverse: 5′-CCA​TGG​TGA​GCA​AAC​ACT​TG-3′), *Il9r* (forward 5′-TCC​TGG​TTC​CTG​ATC​TAC​AGC-3′, reverse 5′-TGT​GTT​TGA​TTT​CAG​TCA​CCT​GG-3′), *Il1rl1* (forward 5′-CTC​TGC​CCG​ACG​TTC​TTG​A-3′, reverse 5′-AAC​CCC​TGA​TGT​GTC​TCA​G-3′), *Gata3* (forward: 5′-GTC​ATC​CCT​GAG​CCA​CAT​CT-3′, reverse: 5′-AGG​GCT​CTG​CCT​CTC​TAA​CC-3′), *Maf* (forward: 5′-GCA​TGC​TGG​ACA​TGT​ATG​GT-3′, reverse: 5′-ATG​TAC​AAC​GGG​AGG​CTG​AA-3′), and *Bcl6* (forward: 5′-CCG​GCA​CGC​TAG​TGA​TGT​T-3′, reverse: 5′-TGT​CTT​ATG​GGC​TCT​AAA​CTG​CT-3′). Gene expression was normalized to the housekeeping gene *Hprt1* (forward: 5′-GAT​ACA​GGC​CAG​ACT​TTG​TTG​G-3′, reverse: 5′-CAA​CAG​GAC​TCC​TCG​TAT​TTG​C-3′), *Hprt1* (Mm00446968_m1), or *Actb* (forward: 5′-CTA​AGG​CCA​ACC​GTG​AAA​AG-3′, reverse: 5′-ACC​AGA​GGC​ATA​CAG​GGA​CA-3′).

### *In vitro* stimulation and cytokine measurement

Purified ILC2s (live, CD45^+^, Lin^−^, NK1.1^−^, CD127^+^, and KLRG1^+^) were incubated in DMEM with high glucose supplemented with 10% FCS, 10 mM Hepes, 1 mM sodium pyruvate, nonessential amino acids, 80 μM 2-Mercaptoethanol, 2 mM glutamine, 100 U/ml penicillin, and 100 μg/ml streptomycin (all from Gibco) in 96-well U-bottom microtiter plates (Nunc) for 1 or 3 days at 37°C and 5% CO_2_. If indicated, the culture was supplemented with IL-7 alone or with IL-7 and IL-25, or IL-7 and IL-33, or IL-7 and TSLP (BioLegend, 20 ng/ml, each).

Cytokine concentration in culture supernatants were determined by using a customized LEGENDplex multiplex beads-based assay (BioLegend) according to the manufacture’s protocol to detect murine IL-5, IL-9, and IL-13. Samples were recorded on a custom configuration Fortessa flow cytometer and the FACS Diva software (BD Biosciences), and the flow cytometry data files were analyzed using the LEGENDplex cloud-based analysis software suite (BioLegend).

### Western blot

ILC2s were sort purified from the small intestine into RIPA buffer. Cells were lysed by freeze–thaw cycles and centrifuged at 12,000 *g* for 10 min at 4°C to collect supernatants containing total protein lysates. Protein concentrations were determined using Bradford reagent, and equal amounts of protein were loaded per lane onto 12% SDS–PAGE gels and electrophoresed at 150 V. Proteins were transferred to nitrocellulose membranes, which were then blocked with 5% skimmed milk powder (in PBS + 0.05% Tween-20) and incubated overnight with a Blimp-1 primary antibody (Cell Signaling Technology, 1:200). After washing, membranes were incubated with an HRP-conjugated secondary antibody (anti-rabbit HRP; Cell Signaling Technology, 1:500). Signals were visualized using ECL reagent (GE Healthcare).

Membranes were then stripped (0.2 M glycine + 0.05% Tween-20, 75°C) and reprobed with an Actin primary antibody (Cell Signaling Technology, 1:500). Blots were developed as described above. Blimp-1 bands were quantified densitometrically using ImageJ software and normalized to Actin.

### Helminth infection and allergic asthma induction

Third-stage larvae of *N. brasiliensis* were purified with a Baermann apparatus. After washing three times in PBS, larvae were counted, and 500 purified larvae were injected subcutaneously in PBS. Mice were killed, organs were analyzed, and worm burden was determined in the small intestine 7 days after infection or on day 9 and 10, where indicated.

For the 10-day infection model, fecal pellets (at least two pellets per mouse) were collected to determine any changes in the fecundity of infecting worms. Fecal pellets were weighed in 2.0-ml Eppendorf tubes (USA Scientific), softened in saturated NaCl (Sigma-Aldrich) water at the room temperature for an hour, followed by further dissociation by using wide-bore 1,000-μl pipette tips. Dissociated fecal materials were then strained through 100-µm cell strainer into 50-ml conical tubes (Nunc, Thermo Fisher Scientific) and brought to 10 ml final volume with additional saturated NaCl water by also rinsing the strainer. Immediately after mixing thoroughly, an aliquot of strained fecal material solution was transferred into the McMaster Microscope Slides (Eggzamin). The egg numbers were counted and normalized to egg number per mg of feces by using the chamber grid volume (150 μl) of the McMaster slide, the final volume of fecal material solution (10 ml), and the weight (in mg) of the fecal pellets for each mouse.

For allergic asthma induction, 30 μg of Papain (Roche) in PBS were administered intranasally on three consecutive days. Mice were killed 7 days after initial administration, organs were collected and analyzed.

### *In vivo* cytokine treatments

For examination of *in vivo* changes in the Blimp-1 expression in ILC2s, 12 μg/kg of recombinant murine IL-33 (carrier-free, R&D Systems) in sterile PBS or PBS only vehicle control was administered intraperitoneally (i.p.) into Blimp-1–YFP transgenic mice daily for 3 days, and the mice were killed on day 4. Single-cell suspension isolated from the harvested lungs and mLN tissues were used to assess the level of Blimp-1–YFP reporter expression in ILC2s by flow cytometry.

### Bulk RNA-seq and analysis

ILC2s were sort-purified as Sytox blue^−^ CD45^+^ lineage (CD3, CD5, CD19, and Ly6G)^−^ NK1.1^−^ CD127^+^ KLRG1^+^ from the small intestine of *Nmur1*^Cre^*Prdm1*^flox/flox^ and *Prdm1*^flox/flox^ littermate control mice. Further, ILC2s from small intestine were sort purified from *Il17rb*^−/−^, *Il33*^−/−^, *Il1rl1*^−/−,^*Crlf2*^*−/−*^, *Irf4*^−/−^, and WT mice in steady state and from *Il17rb*^−/−^, *Il33*^−/−^, *Crlf2*^*−/−*^, and WT mice on day 7 after *N. brasiliensis* infection. Cells were sorted into Trizol, and RNA was isolated using the RNeasy microRNA kit (Qiagen) according to the protocol provided by the manufacturer. RNA-seq libraries were prepared by the Max Delbrück Center for Molecular Medicine Berlin Institute for Medical Systems Biology (MDC BIMSB) Core Bioinformatic Facility using the SMARTer Stranded Total RNA-Seq Kit–Pico (Takara). Sequencing was performed on a NovaSeq 6000 (Illumina), yielding 100-bp single-end reads. RNA-seq reads were mapped to the mouse genome (mm10) with STAR (Dobin et al., 2012) version 2.7.3a using default parameters. Reads were assigned to genes with FeatureCounts (Liao et al., 2013) with the following parameters: -t exon -g gene_id. The differential expression was carried out with DESeq2 version 1.22.1 (Love et al., 2014) using default parameters. We kept genes with a minimum baseMean of 50.

### scRNA-seq

ILC2s from mLNs of *Nmur1*^Cre^*Prdm1*^fl/fl^ and *Prdm1*^fl/fl^ littermate control mice were isolated as described on day 7 after *N. brasiliensis* infection. Cells were sort purified as live CD45^+^ Lin^−^, NK1.1^−^, and KLRG1^+^ into PBS. Cells were validated for integrity, and scRNA-seq libraries were generated according to the Chromium Next GEM Single Cell 3ʹ Reagent Kits version 3.1 User Guide (CG000204) by 10x Genomics. Briefly, a droplet emulsion was generated in a microfluidic chip followed by barcoded cDNA generation inside the droplets. Purified and amplified cDNA was then subjected to library preparation and sequenced on a NovaSeq 6000 instrument (Illumina) to a minimal depth of 40,000 mean reads per cell. Raw sequence reads were processed using Cell Ranger (version 5.0.0), including the default detection of intact cells. Mkfastq and count were used in default parameter settings for demultiplexing and quantification of gene expression. Refdata-cellranger- mm10–1.2.0 was used as reference. Single-nucleus RNA-seq data alignment and gene expression quantification was carried out with Cell Ranger (version 5.0.0), using as a reference refdata-gex-mm10-2020-A mouse genome. Cell Ranger output was analyzed with R (version 4.4.1) using the Seurat package (version 5.2.1) (Hao et al., 2024). All analyses were performed using a filtered feature barcode matrix. Genes expressed in <3 cells and cells expressing <200 genes or >5,000 genes were excluded, as well as cells with over 10% mitochondrial reads. Seurat objects from Ctrl and cKO samples were merged. Counts were normalized using the NormalizeData function, and variable features were identified using FindVariableFeatures (method = “vst,” nfeatures = 2,000). Data were scaled and centered using the ScaleData function, and dimensionality reduction was performed using PCA (selecting the top 20 principal components). The two layers were integrated using the IntegrateLayers function with the HarmonyIntegration method. Clusters were identified using the FindNeighbors and FindClusters functions with a resolution of 0.5, resulting in 13 clusters (harmony_clusters). Data visualization was performed using the uniform manifold approximation and projection algorithm. Non-ILC2 clusters were excluded, resulting in 9 clusters for further analysis. Differential gene expression analysis per cluster was conducted using the FindAllMarkers function with default parameters and FindMarkers for pairwise comparison between Ctrl and cKO samples.

### Bulk ATAC-seq

Bulk ATAC-seq was performed on FACS-sorted cells using a standard transposition protocol. Briefly, ILC2s were sort-purified as Sytox blue^−^ CD45^+^ lineage (CD3, CD5, CD19, and Ly6G)^−^ NK1.1^−^ CD127^+^ KLRG1^+^ from the small intestine of *Nmur1*^Cre^*Prdm1*^fl/fl^ and *Prdm1*^fl/fl^ littermate control mice. Cells were lysed in a nonionic detergent buffer (10 mM Tris-HCl, pH = 7.4, 10 mM NaCl, 3 mM MgCl_2_, and 0.1% NP-40) to isolate nuclei, which were immediately subjected to transposition with Tn5 transposase (Nextera DNA Library Prep, Illumina) in tagmentation buffer at 37°C. Following transposition, DNA was purified using AMPure XP beads according to the manufacturer’s instructions. To determine the appropriate number of amplification cycles, an initial qPCR was performed on a small aliquot of tagmented DNA using indexed primers, and the remaining material was amplified for library preparation based on the observed Ct value. Amplified libraries were then purified a second time with AMPure XP beads to remove primer dimers and small fragments. Final libraries were assessed for size distribution and concentration, and sequencing was performed on a NovaSeq X Plus (Illumina), yielding 100-bp paired-end reads.

The reads were processed as following: initial quality assessment was performed using FastQC version 0.12.1, and results were summarized using MultiQC version 1.27 (Ewels et al., 2016). Adapter trimming and removal of low-quality bases were performed with Cutadapt version 5.2 (Martin, 2011), and trimmed reads shorter than 20-bp were discarded. High-quality reads were aligned to the mouse reference genome (mm10) using Bowtie2 version 2.5.4 (Langmead et al., 2009). Postalignment filtering removed reads mapping to mitochondrial DNA, low-quality alignments (MAPQ <30), and improperly paired fragments. BAM file manipulation and filtering were conducted with BamTools 2.31.1, and PCR duplicates were identified and removed (Barnett et al., 2011). Fragment size distributions were then inspected to confirm expected nucleosome-free, mono-, and di-nucleosomal fragment patterns. Peak calling was performed with MACS2 using ATAC-appropriate parameters (--nomodel, shift = −100, extend = 200; (Feng et al., 2012; Zhang et al., 2008). Normalized coverage tracks (bigWig) were generated for visualization and global chromatin accessibility patterns. Locus-specific visualization of multivariate genomic signals was performed using IGV (Robinson et al., 2011). For differential accessibility analysis, reads overlapping consensus peak regions were quantified using Bedtools (Quinlan and Hall, 2010), and count matrices were subjected to statistical analysis using DESeq2 DESeq2 version 1.22.1 to identify differentially accessible regions between experimental conditions.

### Histology and immunofluorescence microscopy

Small intestine from *N. brasiliensis*–infected animals was fixed in 4% paraformaldehyde at 4°C until tissue embedding. Paraffin-embedded sections were de-paraffinized and rehydrated. Sections were permeabilized with 0.5% Triton-X in PBS and blocked with PBS 0.5% Triton X-100 and 10% serum and stained. Rabbit anti-Dclk1 (Abcam) followed by donkey anti-rabbit antibody coupled to Alexa Fluor 555 (Thermo Fisher Scientific) were applied, and nuclei were counterstained with DAPI (Thermo Fisher Scientific). Images were captured on a Zeiss Axio Observer 7 microscope and analyzed with Zen software (Zeiss). For tuft cell numbers, three representative villi were counted on five independent images per mouse using ImageJ.

### *Ex vivo* measurement of cytokine production

To detect intracellular cytokine levels *ex vivo*, isolated single-cell suspensions were stimulated with 100 ng/ml phorbol 12-myristate 13-acetate (PMA) (Sigma-Aldrich) and 1 μg/ml ionomycin (Sigma-Aldrich) for 4 h in the presence of 10 μg/ml brefeldin A (Sigma-Aldrich) in complete RPMI-1640 medium (containing 10% FBS, 55 µM 2-mercaptoethanol, 5 mM HEPES, 2 mM L-glutamine [GIBCO], 1 mM sodium pyruvate [GIBCO], 100 µM nonessential amino acids [GIBCO], 100 U/ml penicillin, and 100 μg/ml streptomycin [Corning]), followed by surface marker staining on ice in FACS buffer supplemented with 2% normal mouse serum (Jackson ImmunoResearch). Intracellular cytokine staining was performed using the BD Cytofix/Cytoperm Fixation and Permeabilization kit (BD Biosciences). Cytokines were detected by staining for IL-5 (TRFK5) and IL-13 (eBio13A) and analyzed on a 5 laser, 18 color custom-configuration BD LSRFortessa (BD).

### Statistical analysis

Data are plotted showing the mean ± SD. P values of data sets were determined by unpaired two-tailed Student’s *t* test, ordinary one-way ANOVA with Tukey’s multiple comparisons test, or two-way ANOVA with Šidák’s multiple comparisons test, with 95% confidence interval. Normal distribution was assumed. Statistical tests were performed with Graph Pad Prism version 9 software (GraphPad Software, Inc.). *P < 0.05; **P < 0.01; ***P < 0.001; ****P < 0.0001; and ns, not significant.

### Online supplemental material

Fig. S1 shows the gating strategy for intestinal ILC2 sorting, pathway analysis of bulk RNA-seq data, and expression of Regnases and *Prdm1* in ILC2s, as well as phenotypic characterization of *Prdm1*- and *Zc3h12c*-deficient ILC2s. Fig. S2 provides a more detailed transcriptomic analysis of ILC2s from *N. brasiliensis*–infected KO mice (*Il33*^−/−^, *Il1rl1*^−/−^, *Il17rb*^−/−^, and *Crlf2*^−/−^) compared with WT controls, including differential gene expression and pathway enrichment analysis. Fig. S3 shows gating and flow cytometry analysis of lymphoid and myeloid compartments in *Prdm1*- and *Zc3h12c-*deficient mice during *N. brasiliensis* infection. Fig. S4 shows scRNA-seq analysis of mLN ILC2s, defining cluster identity, marker expression, and functional pathway enrichment, together with violin plots for specific markers. Fig. S5 shows gating and flow cytometry analysis of *Prdm1*-deficient ILC2s after cytokine stimulation and papain challenge. PCA of bulk ATAC-seq and heatmaps of top-regulated genes in *Irf4*^−/−^ and WT ILC2s, compared with different bulk RNA-seq datasets, are provided, revealing shared transcriptional programs between *Prdm1*- and *Irf4*-deficient ILC2s.

## Supplementary Material

SourceData F2 is the source file for Fig. 2.

## Data Availability

The RNA-seq data are deposited in the Sequence Read Archive repository database under the accession number PRJNA1247146. Source data are provided with this manuscript. Data were analyzed using the standard Seurat 5.2.1 pipeline or with the stated variations. User scripts will be shared upon request.
