# Supplementary figures and images for: Blimp-1 integrates alarmin signals in ILC2s and drives proinflammatory functions required for type 2 immunity
Source: J Exp Med. 2026 Mar 31;223(5):e20250781. doi: 10.1084/jem.20250781 (PMC13037585; doi:10.1084/jem.20250781)

**K**

Protein ladder (KDa)

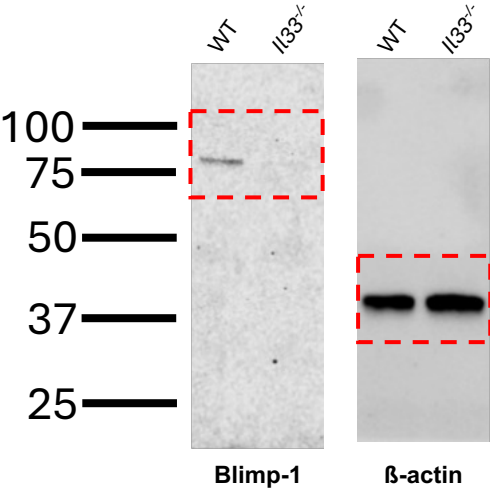

**L**

Protein ladder (KDa)

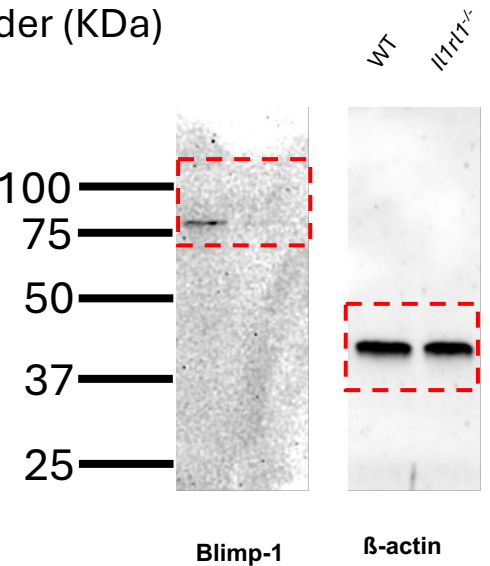

Supplement: SourceData F2 — is the source file for Fig. 2. [file jem_20250781_sourcedataf2.pdf]
